# Reactive oxygen species drive evolution of pro-biofilm variants in pathogens by modulating cyclic-di-GMP levels

**DOI:** 10.1098/rsob.160162

**Published:** 2016-11-23

**Authors:** Song Lin Chua, Yichen Ding, Yang Liu, Zhao Cai, Jianuan Zhou, Sanjay Swarup, Daniela I. Drautz-Moses, Stephan Christoph Schuster, Staffan Kjelleberg, Michael Givskov, Liang Yang

**Affiliations:** 1 Lee Kong Chian School of Medicine, Nanyang Technological University, Singapore 639798; 2 School of Biological Sciences, Nanyang Technological University, Singapore 639798; 3 Singapore Centre for Environmental Life Sciences Engineering (SCELSE), Nanyang Technological University, Singapore 637551; 4 Interdisciplinary Graduate School, Nanyang Technological University, Singapore 637551; 5 Integrative Microbiology Research Centre, South China Agricultural University, Guangzhou 510642, People's Republic of China; 6 Department of Biological Sciences, National University of Singapore, Singapore 117543; 7 NUS Environmental Research Institute, National University of Singapore, Singapore; 8 Center for Marine Bio-Innovation and School of Biotechnology and Biomolecular Sciences, University of New South Wales, Sydney 2052, Australia; 9 Costerton Biofilm Center, Department of Immunology and Microbiology, University of Copenhagen, 2200 København N, Denmark

**Keywords:** biofilms, c-di-GMP, rough small colony variants, reactive oxygen species, *Pseudomonas aeruginosa*, adaptive evolution

## Abstract

The host immune system offers a hostile environment with antimicrobials and reactive oxygen species (ROS) that are detrimental to bacterial pathogens, forcing them to adapt and evolve for survival. However, the contribution of oxidative stress to pathogen evolution remains elusive. Using an experimental evolution strategy, we show that exposure of the opportunistic pathogen *Pseudomonas aeruginosa* to sub-lethal hydrogen peroxide (H_2_O_2_) levels over 120 generations led to the emergence of pro-biofilm rough small colony variants (RSCVs), which could be abrogated by l-glutathione antioxidants. Comparative genomic analysis of the RSCVs revealed that mutations in the *wspF* gene, which encodes for a repressor of WspR diguanylate cyclase (DGC), were responsible for increased intracellular cyclic-di-GMP content and production of Psl exopolysaccharide. Psl provides the first line of defence against ROS and macrophages, ensuring the survival fitness of RSCVs over wild-type *P. aeruginosa*. Our study demonstrated that ROS is an essential driving force for the selection of pro-biofilm forming pathogenic variants. Understanding the fundamental mechanism of these genotypic and phenotypic adaptations will improve treatment strategies for combating chronic infections.

## 1. Background

Bacterial pathogens can colonize human hosts for years and cause persistent/chronic infections [1,2]. These infections predominantly result from biofilm formation, whereby secreted extracellular-polymeric substances (EPS) such as adhesive proteins, biosurfactants, extracellular DNA and exopolysaccharides act as physical barriers to protect bacterial cells from the host immune clearance and antimicrobial treatments [3–5].

The transition between free-living planktonic and sessile biofilm lifestyles of most bacterial species is mediated by bis-(3′-5′)-cyclic dimeric guanosine monophosphate (c-di-GMP), a global intracellular secondary messenger [6,7]. Increase in intracellular c-di-GMP content is caused by the activity of diguanylate cyclases (DGCs) [8,9], resulting in biofilm formation, whereas phosphodiesterases (PDEs) degrade c-di-GMP [10,11] and cause biofilm dispersal to the planktonic phase [12,13]. C-di-GMP signalling is involved not only in motility, surface attachment and production of EPS [14–16], but also in the sensing of chemicals and surfaces [8]. C-di-GMP signalling also regulates virulence and stress responses in pathogens [17,18].

*Pseudomonas aeruginosa* is the leading cause of chronic lung infection and morbidity of patients with cystic fibrosis (CF) [19,20]. It is able to survive and form biofilms despite the presence of a functional host immune system, antibiotic treatments and competing pathogens, such as *Staphylococcus aureus* in the CF lung environment [21–24]. Investigations of adaptive evolution of *P. aeruginosa* in CF lung infections have provided valuable information for our current understanding of chronic infections. *P. aeruginosa* can colonize the CF lungs for decades and usually gives rise to pro-biofilm sub-populations after adaptive evolution [25,26]. The occurrence of rough small colony variants (RSCVs) and mucoid strains is often reported from chronic CF infections, implying these variants have better fitness than their ancestors [27,28].

Although the characteristics of RSCVs have been studied [29,30], the stimuli and mechanisms leading to the evolution of such adapted sub-populations are unclear. Given the CF environment consists of oxidative stress, high antibiotic concentrations, high pro-inflammatory cytokine levels and poor nutrient conditions [31–35], it is highly likely that each of the CF-derived environmental factors can drive the adaptive evolution of pathogens differently.

Experimental evolution assays have been used in previous studies to investigate bacterial adaptation to various conditions such as antibiotic treatments and carbon sources [18,36]. Here, we employed the adaptive experimental evolution assay to evolve *P. aeruginosa* against an important host-derived antimicrobial, reactive oxygen species (ROS), resulting in the occurrence of RSCVs with a strong capability for biofilm formation and ROS stress resistance.

The RSCVs isolated from H_2_O_2_ treated cultures showed increased intracellular c-di-GMP content. Next-generation sequencing (NGS) analysis revealed that *wspF* mutation was associated with these isolated RSCVs. The *wspF* is part of the chemosensory-like system Wsp (wrinkly spreader phenotype) [8], whose product acts as a repressor against WspR (DGC), thus its mutagenesis leads to the de-repression of WspR. The increased production of exopolysaccharides (especially Psl) as the result of *wspF* mutation conferred resistance of RSCVs to H_2_O_2_ treatment. Hence, this work strongly suggests that exposure to ROS imposes a strong selective pressure on *P. aeruginosa* during chronic colonization and accounts for the occurrence of RSCVs in clinical isolates obtained from CF patients.

## 2. Material and methods

### 2.1. Bacterial strains, plasmids, media and growth conditions

*Escherichia coli* DH5α strain was used for standard DNA manipulations. Luria-Bertani (LB) medium was used to cultivate *E. coli* strains. Batch cultivation of *P. aeruginosa* strains was carried out at 37°C in ABTG (ABT minimal medium supplemented with 5 g l^−1^ glucose) or ABTGC (ABT minimal medium supplemented with 2 g l^−1^ glucose and 2 g l^−1^ casamino acids). For plasmid maintenance in *E. coli*, the medium was supplemented with 100 µg ml^−1^ ampicillin (Ap), 15 µg ml^−1^ gentamicin (Gm) and 15 µg ml^−1^ tetracycline (Tc). When appropriate, the marker selection in *P. aeruginosa*, 30 µg ml^−1^ Gm, 50 µg ml^−1^ Tc or 200 µg ml^−1^ carbenicillin (Cb), was used. The bacterial strains and plasmids are listed in the electronic supplementary material, table S1.

### 2.2. Imaging colony morphology of *Pseudomonas aeruginosa*

Cultures were streaked on LB agar together with 40 µg ml^−1^ Congo Red and 15 µg ml^−1^ Coomassie Brilliant Blue R at 37°C for 48 h as previously described [37]. Colony images were captured by the stereomicroscope (Zeiss Discovery V8, Germany) and processed using IMARIS software (Bitplane AG, Zurich, Switzerland).

### 2.3. Evolution assay of *Pseudomonas aeruginosa* PAO1 in H_2_O_2_ and GSH

Cultures were cultivated overnight from ancestral PAO1 at 37°C, 200 rpm in LB with three biological replicates. Each biological replicate with initially identical populations was then divided into three technical replicates, each grown in ABTGC only or ABTGC with 2 mM H_2_O_2_ + 5, 2.5, 1, 0.25, 0.125 or 0 mM GSH. One per cent of each replicate population was transferred to a new tube of fresh ABTGC with and without 2 mM H_2_O_2_ + GSH at 37°C, 200 rpm for 12 h, allowing each population to experience an estimated 6.67 generations every passage. This was then repeated for 15 days, so that there were an estimated 120 generations of cells grown in 2 mM H_2_O_2_.

The populations were then cryopreserved with 50% glycerol (cryoprotectant) for revival at a later time. To observe the emergence of unique phenotypes arising from treatment with 2 mM H_2_O_2_, the populations were grown on LB agar plates at 37°C overnight. Ten RSCV isolates were identified from the different replicates and frozen with 50% glycerol.

### 2.4. H_2_O_2_ resistance assay

The *P. aeruginosa* cultures were grown overnight and prepared by adjusting the optical density to OD_600_ = 0.3 in ABTGC with and without 4 mM H_2_O_2_. The cultures were grown at 37°C, 200 rpm for 4 h. The cell culture was serially diluted and plated on LB agar plates. The culture plates were incubated in 37°C for 16 h. The numbers of colonies were counted for tabulation of log_10_ CFU ml^−1^. The log_10_ CFU ml^−1^ is calculated by log_10_(average number of colonies × dilution factor × volume used to spread on LB agar plate). Three independent experiments were performed in triplicate, one-way ANOVA and Student's *t*-tests were used to determine statistical significance and the results were shown as the mean ± s.d.

### 2.5. Competitive mixed-treatment assay of rough small colony variant isolates and PAO1

The evolved RSCVs and PAO1 cultures were prepared by adjusting the optical density to OD_600_ = 0.5 in ABTGC. Each RSCV was mixed at equal volumes (1 : 1 ratio) with PAO1 in ABTGC with and without 4 mM H_2_O_2_ (1× MIC). The cultures were grown at 37°C, 200 rpm. Twenty microlitre samples were collected after a 4 h incubation. They were diluted serially in 0.9% NaCl and 100 µl of cell culture was cultivated on LB agar plates for 16 h at 37°C. The RSCVs and PAO1 were differentiated by the morphology of the colonies. The number of colonies was counted for tabulation of CFU ml^−1^. The competition ratio was then calculated by CFU ml^−1^ of RSCV/CFU ml^−1^ of PAO1. Three independent experiments were performed in triplicate, one-way ANOVA and Student's *t*-tests were used to determine statistical significance and the results were shown as the mean ± s.d.

### 2.6. DNA sequencing of rough small colony variants isolates

Individual colonies of the ancestral PAO1, two random PAO1 isolates evolved in ABTGC only and 10 RSCV isolates were first streaked on the LB agar plates and incubated at 37°C for 16 h. Single colonies were picked by a 1 µl inoculation loop and grown in LB at 37°C, 200 rpm for 16 h. Genomic DNA of the *P. aeruginosa* strains was purified using QIAamp DNA Mini Kit (Qiagen, Venlo, The Netherlands). The quality control tests included NanoDrop (Thermo Fisher Scientific) and Qubit (Thermo Fisher Scientific) to ensure the concentration and quality of DNA, and DNA electrophoresis (1% agar) to determine DNA integrity.

The genomic DNA was then sequenced on an Illumina (San Diego, CA, USA) MiSeq V3 platform, generating 300 bp long paired-end reads using the method described in Chua *et al.* [38]. The average insert sizes were 490–544 bp, and average genomic coverage depths were 40–186 fold. Nucleotide differences were generated from the CLC Genomics Workbench 8.0 (CLC bio, Aarhus, Denmark). Briefly, adapters and low quality reads were trimmed. Paired-end reads in FASTQ format for RSCV and control genomes were mapped against the *P. aeruginosa* PAO1 genome (NC_002516). Both the mappings of RSCV and control strains were compared with their ancestor PAO1, and variants were detected using the quality based variant detection method with the required frequency of 35%.

### 2.7. Measurement of mutation rates

PAO1 was grown in ABTGC with or without 2 mM H_2_O_2_ until stationary phase, and plated on LB agar plates with and without 0.78 µg ml^−1^ ciprofloxacin, using the method described in Mandsberg *et al.* [39]. The mutated colonies cultured on LB agar with ciprofloxacin were enumerated to account for point mutations on the *gyr* gene that can easily develop in *P. aeruginosa* exposed to ciprofloxacin [40]. The mutation rate was then calculated by dividing the number of mutations by the final CFU of cells grown on LB agar plates without ciprofloxacin. Three independent experiments were performed in triplicate, the Student's *t*-test was used to determine statistical significance and the results were shown as the mean ± s.d.

### 2.8. The p*_cdrA_-gfp* reporter assay

The cultures of PAO1 with p*_cdrA_-gfp* reporter fusion were grown in ABTGC with or without H_2_O_2_. In total, 200 µl of cell culture was then transferred into each well of a 96-well plate (triplicates). The OD_600_ and GFP fluorescence (excitation 485 nm/emission 535 nm) were measured using the Tecan Infinite 200 microplate reader (Tecan, Austria). The relative fluorescence intensity count was calculated by dividing GFP values by OD_600_ values. Three independent experiments were performed in triplicate, one-way ANOVA and Student's *t*-tests were used to determine statistical significance and the results were shown as the mean ± s.d.

### 2.9. Quantification of pyoverdine

The cultures were grown in ABTGC with or without H_2_O_2_. In total, 200 µl of cell culture was then transferred into each well of a 96-well plate (triplicates). As previously described [13], the OD_600_ and pyoverdine fluorescence (excitation 400 nm/emission 460 nm) were measured using the Tecan microplate reader, and the relative fluorescence intensity count calculated by dividing pyoverdine fluorescence values by OD_600_ values. Three independent experiments were performed in triplicate, one-way ANOVA and Student's *t*-tests were used to determine statistical significance and the results were shown as the mean ± s.d.

### 2.10. C-di-GMP quantification by liquid chromatography–mass spectrometry

Fifteen millilitres of PAO1 cell culture treated with H_2_O_2_, RSCVs, *Burkholderia cenocepacia* strains and *Klebsiella pneumoniae* strains were harvested and washed twice with 1 mM ammonium acetate. An aliquot of cells was used for protein quantification. The remaining cells were lysed in 1 ml acetonitrile/methanol/ddH_2_O (v/v ratio 40 : 40 : 20) using a probe tip ultrasonicator (amplitude 30%; 5 s on, 5 s off) for 1 min on an ice slurry. The cell debris was removed by centrifuging at 13 000*g*, 4 °C for 3 min, rinsing twice. The supernatant containing the nucleotides was lyophilized with the vacuum concentrator. The lyophilized nucleotides were resuspended in 100 µl 1 mM ammonium acetate. The c-di-GMP standard was also used as a reference to identify the c-di-GMP peak and the concentration of c-di-GMP in the samples.

For the detection and quantification of c-di-GMP, a Thermo Accela 1250 series LC system fitted with EQuanMax autosampler and a Thermo Velos Pro Orbitrap mass spectrometer (Thermo Fisher Scientific) were used. Chromatographic separation was achieved using a Nucleodur C18 Pyramid (2 mm × 50 mm, 3 µm) column (Macherey-Nagel GmbH, Düren, Germany) at 40°C, with a solvent flow rate of 0.3 ml min^−1^ and an injection volume of 10 µl. Buffer A was 10 mM ammonium acetate buffer, containing 0.1% acetic acid, and buffer B was acetonitrile, containing 0.1% acetic acid. Solvent gradient conditions were as follows: 0% B from 0 to 3 min; 10% B at 3 min; 90% from 4th to 5th min; 0% B at 5.5th min and equilibrated for 4.5 min. Total run time was 10 min.

Detection was carried out in positive ion electrospray ionization (ESI+) mode. The heater and capillary temperatures were 300°C. Sheath, auxiliary and sweeper gas flows were 40, 15 and 1 arb. units, respectively. Source voltage was 3.5 kV. For quantitation, scan type in selected ion monitoring mode was used at high-resolution (60 000), with an AGC target of 1 × 10^6^. Quantification was achieved via an MS/MS experiment using collision induced dissociation (CID) with normalized collision energy 20% (of maximum), with isolation width of 1 Da and activation time of 30 ms.

To quantify protein concentration, a small aliquot of cell culture was lysed in 100 µl 5 M sodium hydroxide at 95°C for 5 min. The protein concentration was then measured using a Qubit® 2.0 fluorometer (Invitrogen, Thermo Fisher Scientific, CA). The final concentration of c-di-GMP was then normalized with protein quantity. Three independent experiments were performed in triplicate, one-way ANOVA and Student's *t-*tests were used as to determine statistical significance and results were shown as the mean ± s.d.

### 2.11. Psl staining by fluorescent concanavalin-A

Planktonic MiniTn7-*gfp*-tagged cells were grown in ABTGC + 0, 0.5, 1 and 2 mM H_2_O_2_ at 37°C, 200 rpm for 4 h. Additionally, PAO1, mutants and evolved RSCVs with tagged MiniTn7-*gfp* were grown until stationary phase in ABTGC overnight at 37°C, 200 rpm. Ten microlitres of cell culture were then transferred to the glass slide and stained by 5 µM concanavalin-A Alexa Fluor^®^ 647 conjugate (Thermo Fisher Scientific, Cat. No. C21421) [41].

To monitor fluorescence of GFP and Psl stain, the cells were imaged using an LSM780 confocal laser scanning microscope (CLSM; Carl Zeiss, Germany) with 40× objective or 63× oil objective (for planktonic cells) and the images were processed using IMARIS software (Bitplane AG, Zurich, Switzerland). Three independent experiments were performed in triplicate and representative images were shown.

### 2.12. Arabinose-inducible expression of Psl by PAO1/p*_BAD_*-*psl*

A starting culture of PAO1/p*_BAD_-psl* cells containing an l-arabinose-inducible promoter for *psl* operon expression was grown in ABTGC with increasing concentrations of l-arabinose at 37°C, 200 rpm for 3 h until OD_600_ = ∼0.3 was established, as described in Irie *et al.* [42]. The cultures were then treated with and without 4 mM H_2_O_2_ and continued to be grown at 37°C, 200 rpm for an additional 4 h. The cell culture was serially diluted and plated on LB agar plates. The culture plates were incubated at 37°C for 16 h. The colonies were counted for tabulation of CFU ml^−1^. Three independent experiments were performed in triplicate, one-way ANOVA and Student's *t-*tests were used as statistical tests and the results were shown as the mean ± s.d.

### 2.13. Biofilm quantification of PAO1/p*_BAD_*-*psl* by crystal violet assay

The PAO1/p*_BAD_-psl* biofilms were grown in 1 ml ABTGC with increasing concentrations of l-arabinose at 37°C in 24-well plates (Nunc, Denmark). The biofilms were washed three times with 0.9% NaCl and stained with 0.1% crystal violet for 15 min. Excess crystal violet was washed off with 0.9% NaCl. The stained biofilms were dissoluted by 100% ethanol and their OD_595_ values were quantified using a Tecan Infinite 200 microplate reader (Tecan, Austria). Three independent experiments were performed in triplicate, one-way ANOVA and Student's *t-*tests were used to determine statistical significance and the results were shown as the mean ± s.d.

### 2.14. Cellulase treatment of *Pseudomonas aeruginosa* cultures to degrade Psl

The *P. aeruginosa* cultures were grown overnight and prepared by adjusting the optical density to OD_600_ = 0.1 in ABTGC with 1 mg ml^−1^ cellulase (Sigma-Aldrich, Cat. No. C9748) [42]. The cultures were grown to OD_600_ = ∼0.3 at 37°C, 200 rpm for 3 h. The cultures were treated with 0 or 4 mM H_2_O_2_ and further grown at 37°C, 200 rpm for 4 h. The cell culture was serially diluted and plated on LB agar plates. The culture plates were incubated in 37°C for 16 h. Colonies were counted for tabulation of CFU ml^−1^. Three independent experiments were performed in triplicate. One-way ANOVA and Student's *t-*tests were used to determine statistical significance and the results were shown as the mean ± s.d.

### 2.15. RAW264.7 macrophages culture

The murine RAW264.7 macrophage cell lines (ATCC No. TIB-71) were cultivated in Dulbecco's modified Eagle's medium (DMEM) (Life Technologies, Thermo Fisher Scientific), supplemented with 10% fetal bovine serum (FBS) (Gibco™, Thermo Fisher Scientific). Cell cultures were incubated in 75 cm^2^ cell culture flasks at a density of 1.0 × 10^6^ cells ml^−1^ at 37°C, 5% CO_2_ for 72 h.

### 2.16. Macrophage phagocytosis assay

As previously described [12], 5 × 10^5^ macrophages were grown in each well of a 24-well culture plate (Nunc, Denmark). Macrophages were infected with bacterial suspension at a multiplicity of infection (MOI) of 100 : 1. The co-cultures were incubated at 37°C and 5% CO_2_ for 2 h. The macrophages were washed three times with phosphate-buffered saline (PBS) to remove extracellular bacteria, and then lysed with ddH_2_O containing 0.5% Triton X-100. The cell lysates were then serially diluted, and 100 µl of each dilution was plated on triplicate LB agar plates and incubated overnight at 37°C. The number of colonies was enumerated, and CFU ml^−1^ was tabulated. Experiments were performed in triplicate, one-way ANOVA and Student's *t-*tests were used to determine statistical significance and the results were shown as the mean ± s.d.

### 2.17. Macrophage cytotoxicity assay

RAW264.7 macrophages (5 × 10^5^ ml^−1^ in each well) were grown in 24-well culture plates as previously described [12]. Triplicate cultures were infected with bacterial suspension at a MOI of 100 : 1. Macrophages were incubated for 4 h in 37°C, 5% CO_2_, then 20 µM propidium iodide (PI) was used to stain dead cells and assess macrophage cytotoxicity, leaving live cells unstained. Macrophages stained by PI (excitation 535 nm, emission 617 nm) under epifluorescent microscopy (20× objective) were counted as dead. Macrophages from five replicate images (on average 100 macrophages per image) of each sample were counted, enabling the ratio of dead cells to live cells to be calculated. Three independent experiments were performed in triplicate, one-way ANOVA and Student's *t-*tests were used to establish statistical significance and the results were shown as the mean ± s.d.

## 3. Results

### 3.1. Emergence of rough small colony variants during adaptive evolution of *Pseudomonas aeruginosa* against ROS

To determine if oxidative stress provokes the adaptive evolution of *P. aeruginosa*, *P. aeruginosa* was cultivated in the presence of a sub-lethal concentration of H_2_O_2_ (2 mM, 0.5 × minimal inhibitory concentration (MIC)) for 120 generations. This simulated the presence of ROS produced by polymorphonuclear leucocytes (PMNs) in the CF lung, which generate oxidative stress as a selective pressure on *P. aeruginosa* cells [43]. Although a millimolar range of H_2_O_2_ is required to kill most bacteria and bacteria internalized in the phagosome are usually exposed to micromolar concentrations, the rates of free radical generation within the phagosome are estimated to be 10 000 times faster, leading to higher concentrations of ROS than have been experimentally tested [44]. Hence, the sufficiently high levels of ROS present can be potentially damaging to bacterial cells.

**Figure 1.**
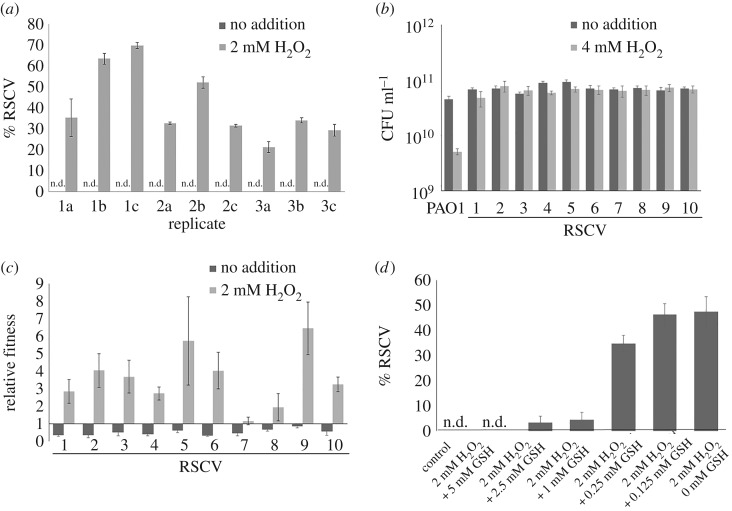
Evolution assay of *Pseudomonas aeruginosa* in H_2_O_2_. (*a*) Percentage of rough small colony variants (RSCVs) after evolution in the presence of H_2_O_2_ compared with the control. (*b*) Resistance of RSCV isolates compared with PAO1 in H_2_O_2_. (*c*) Competitive fitness assay between RSCV isolates and PAO1 grown in H_2_O_2_. (*d*) Antioxidants (glutathione; GSH) reduced the proportion of RSCVs formed with ROS treatment, in a dose-dependent manner. Means ± s.d. from triplicate experiments are shown.

After 120 generations, colonies with RSCV phenotype were observed on the agar plates inoculated with 2 mM H_2_O_2_ treatment (electronic supplementary material, figure S1), while no distinctive phenotypes were observed in the control populations without H_2_O_2_ treatment. The percentage of RSCVs in H_2_O_2_ exposed *P. aeruginosa* populations reached around 40% (figure 1*a*), implying that ROS could significantly contribute to the occurrence of RSCVs. We also showed that *P. aeruginosa* was able to evolve within 120 generations, when compared with a shorter 47.5 generation period in the evolution of RSCVs with antibiotics [18]. As *P. aeruginosa* can persist in CF lungs for decades, spanning 200 000 bacterial generations [33,45], there is more than sufficient time for the metabolically active [46] *P. aeruginosa* to adapt in CF lungs.

The resistance of randomly selected RSCV clones to H_2_O_2_ was then measured. The RSCVs showed up to 16-fold increase in MIC to H_2_O_2_ (table 1) compared with the ancestral PAO1 strain. The RSCV clones also survived better than PAO1 after treatment with 4 mM H_2_O_2_ (1× MIC) (figure 1*b*). Next, we measured the fitness of the selected RSCVs by co-culturing them with ancestral PAO1. In the absence of 2 mM H_2_O_2_, the ancestral PAO1 outcompeted the RSCVs (figure 1*c*), but RSCVs outcompeted PAO1 in the presence of 2 mM H_2_O_2_ (figure 1*d*), confirming that the RSCVs were favoured and selected for in the presence of oxidative stress. Interestingly, RSCV isolates ‘7’ and ‘8’ showed only slightly higher fitness against the PAO1 ancestral strain, compared with the rest of the RSCV isolates, implying different magnitudes of adaptation within the evolved populations.

**Table 1.** MIC of evolved RSCV isolates to H_2_O_2_.

strain/isolate	MIC (mM)
PAO1	4
RSCV 1	32
RSCV 2	64
RSCV 3	64
RSCV 4	64
RSCV 5	64
RSCV 6	32
RSCV 7	64
RSCV 8	64
RSCV 9	8
RSCV 10	64

We determined if the selection of RSCV by oxidative stress could be neutralized by the addition of l-glutathione, a commonly used antioxidant [47]. As expected, l-glutathione reduced the proportion of RSCVs formed in a dose-dependent manner, compared with that in the absence of antioxidants (figure 1*d*).

### 3.2. Increased c-di-GMP content confers resistance of rough small colony variants to H_2_O_2_

**Figure 2.**
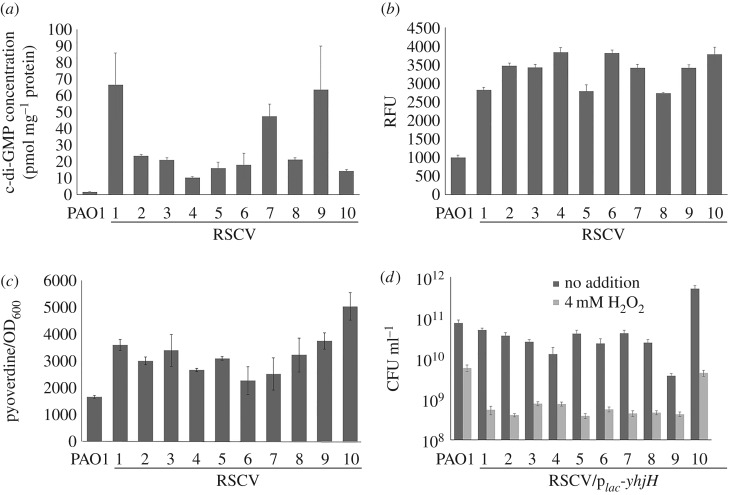
Adaptation of RSCVs to H_2_O_2_ is dependent on induction of c-di-GMP content of isolates. (*a*) C-di-GMP content of RSCVs quantified by HPLC. (*b*) The p*_cdrA_*-*gfp* expression in RSCVs. (*c*) Pyoverdine production in RSCVs. (*d*) Expression of p*_lac_-yhjH* in RSCVs reduces their resistance to H_2_O_2_. Means ± s.d. from triplicate experiments are shown.

As c-di-GMP signalling is implicated in RSCV formation [27], we examined the intracellular c-di-GMP content in the selected RSCV isolates. Liquid chromatography–mass spectrometry (LC-MS) quantification of the whole cell extract showed that the RSCV isolates contained higher intracellular c-di-GMP than wild-type *P. aeruginosa* PAO1 (figure 2*a*). This was in accordance with their higher expression levels of the c-di-GMP reporter fusion p*_cdrA_-gfp* [48,49] (figure 2*b*) and higher pyoverdine production [50,51] (figure 2*c*), compared with that of ancestral PAO1. To confirm that the increased H_2_O_2_ resistance of RSCVs was attributed to enhanced intracellular c-di-GMP content, we transformed a p*_lac_*-*yhjH* plasmid [52] into the RSCVs to deplete their intracellular c-di-GMP. As expected, constitutive expression of the YhjH PDE in the RSCVs attenuated their resistance to H_2_O_2_ (figure 2*d*). These results indicate that c-di-GMP signalling plays an important role in the evolutionary development of PAO1 to RSCVs under ROS stress and corroborated previous studies showing that CF RSCV isolates possessed high c-di-GMP levels [27].

### 3.3. Comparative genomic analysis of rough small colony variants reveal parallel evolution traits

**Figure 3.**
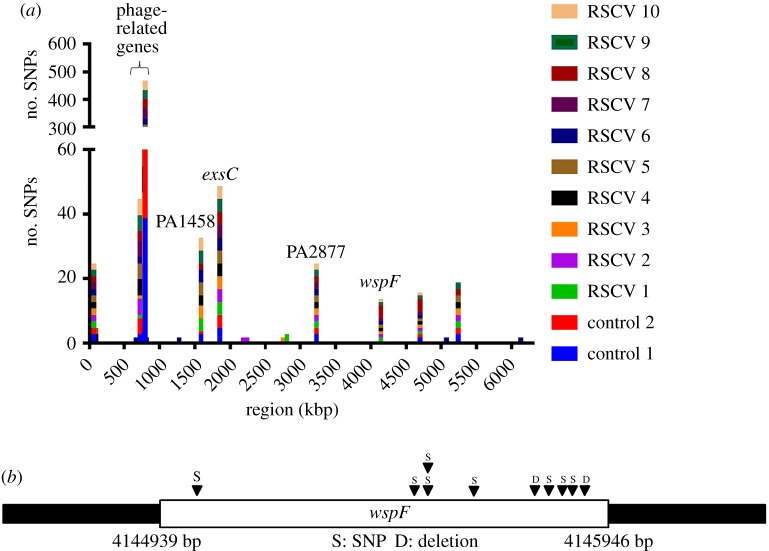
Genetic sequencing for identification of mutation sites. (*a*) Number of mutations identified in evolved genotypes. (*b*) Location of mutations in the *wspF* gene of different RSCVs.

To gain insights into the underlying genetic changes contributing to c-di-GMP-linked RSCV phenotype development with prolonged oxidative stress via c-di-GMP signalling, we sequenced the genomes of 10 randomly selected RSCV isolates and 2 control PAO1 colonies using the Illumina MiSeq platform. Two random control PAO1 colonies were picked from cultures grown in a medium without H_2_O_2_ for 120 generations. A series of point mutations were evident in the sequences of 10 randomly selected RSCVs and control PAO1 colonies, compared with the ancestor PAO1 sequence (electronic supplementary material, tables S2 and S3). The distribution of the identified mutations across the PAO1 genome is illustrated in figure 3*a*. The functional annotation of these RSCV-associated mutations is listed in the electronic supplementary material, tables S2 and S3. Several parallel evolution traits were observed from both RSCVs and control PAO1 colonies, including mutations in *PA0727* and *PA0720* (encoding phage-related genes), *PA1458* (encoding a two component sensor), *exsC* (encoding an anti-anti-activator of the type III secretion system), *PA2877* (encoding a probable transcriptional regulator), intergenic region 4 699 910 bp and intergenic region 5 242 141 bp.

As *P. aeruginosa* commonly accumulate mutations even in planktonic cultures and the mutations are common between control and ROS-evolved strains, the above-mentioned mutations are not deemed to be generated by ROS pressure. We also found that the mutations were not attributed to highly mutagenic growth conditions, as there was no significant increase in mutation rates with ROS treatment (electronic supplementary material, figure S2) compared with control medium, with the mutation rates similar to previous reports [38]. Moreover, the cell populations did not develop mutations in the *mutS* gene, which is important in mismatch repair and its mutation can lead to the emergence of multiple phenotypic variants [53].

Interestingly, there was one parallel evolution trait, *wspF* gene mutation, which could only be detected in the genomes from the RSCVs and not in the control PAO1 colonies. All RSCV genomes had loss of function mutations in the *wspF* gene at different sites (figure 3*b*; electronic supplementary material, table S1). The Δ*wspF* mutation caused the de-repression of the WspR DGC, leading to increased c-di-GMP synthesis and high intracellular c-di-GMP content, resulting in bacterial cell clumping (electronic supplementary material, figure S1) [54]. This indicated that enhanced intracellular c-di-GMP content might play a positive role in *P. aeruginosa'*s adaptation to oxidative stress.

### 3.4. Regulatory roles of *wspF* on ROS-mediated resistance of *Pseudomonas aeruginosa*

**Figure 4.**
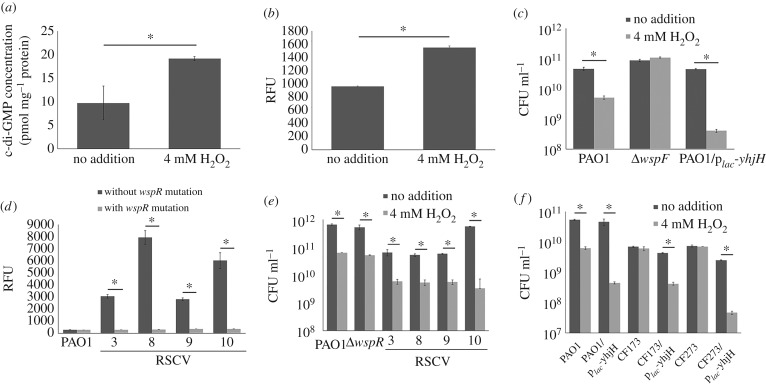
Δ*wspF* mutation is important in the induction of c-di-GMP in the presence of ROS. (*a*) Induction of c-di-GMP in PAO1 by short term (4 h) H_2_O_2_ exposure (LC-MS quantification). (*b*) Induction of p*_cdrA_*-*gfp* expression level in PAO1 by short term (4 h) H_2_O_2_ exposure. (*c*) Resistance of PAO1, Δ*wspF*, PAO1/p*_lac_-yedQ* and PAO1/p*_lac_-yhjH* strains to 4 mM H_2_O_2_. (*d*) p*_cdrA_-gfp* expression of evolved RSCV isolates with epistatic *wspR* mutations to H_2_O_2_. (*e*) Resistance of evolved RSCV isolates with epistatic *wspR* mutations to H_2_O_2_. (*f*) Resistance of PAO1 and clinical RSCV isolates with known *wspF* mutations to H_2_O_2_ treatment. Means ± s.d. from triplicate experiments are shown; *p* < 0.05, one-way ANOVA.

To investigate whether intracellular c-di-GMP content is increased under conditions of ROS stress, we measured the intracellular c-di-GMP content and expression of c-di-GMP reporter fusion p*_cdrA_-gfp* with H_2_O_2_ treatment. Both intracellular c-di-GMP content and p*_cdrA_-gfp* expression levels were increased in PAO1 strain after exposure to 4 mM H_2_O_2_ for 4 h (figure 4*a*,*b*). Given the increase in intracellular c-di-GMP content in adaptation to H_2_O_2_ stress and the presence of *wspF* mutations in our experimental evolution assay, we hypothesized that the genetic basis of adaptation to ROS was directly linked to the mutation in *wspF*, which resulted in the de-repression of *wspR* DGC [8] and a subsequent increased production of c-di-GMP. Hence, we used an isogenic Δ*wspF* mutant as previously described [51] to ascertain ROS resistance. We also used ‘locked’ high and low c-di-GMP content strains of PAO1 by using the p*_lac_-yedQ* and p*_lac_-yhjH* plasmids, respectively. The *yedQ* gene encodes an *E. coli* DGC that synthesizes c-di-GMP [50,55,56], thus the exogenous addition of the p*_lac_-yedQ* would enable the constitutive production of c-di-GMP within the species, resulting in the highly aggregative nature of the colonies (electronic supplementary material, figure S1). The *yhjH* gene encodes an *E. coli* PDE that degrades c-di-GMP [56,57], so the presence of p*_lac_-yhjH* plasmid would cause the reduction of intracellular c-di-GMP levels.

**Figure 5.**
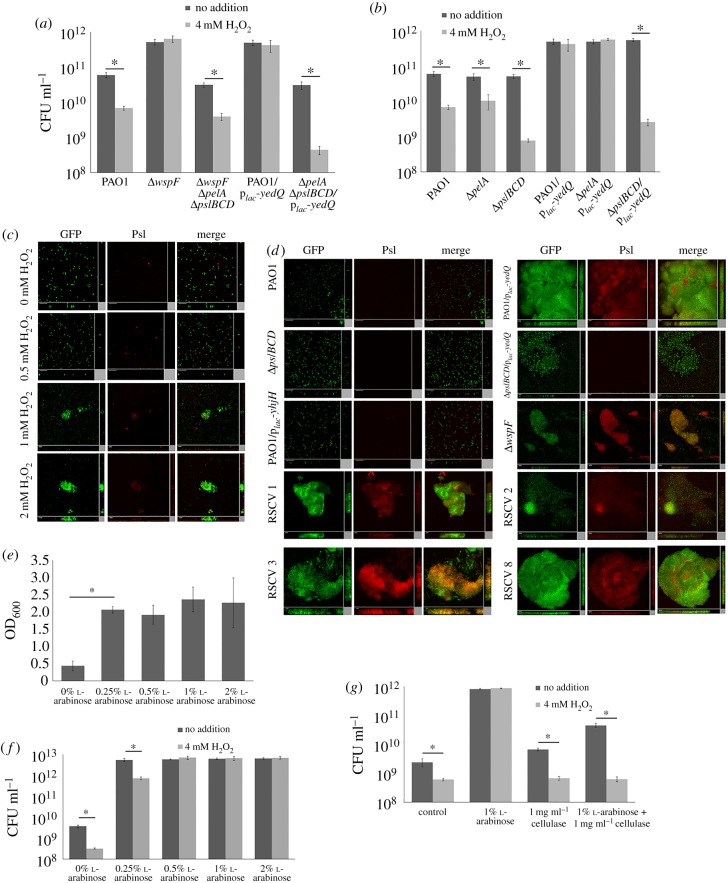
C-di-GMP-mediated exopolysaccharides were required for resistance to ROS stress. (*a*) Resistance of PAO1, Δ*wspF*Δ*pelA*Δ*pslBCD* and Δ*pelA*Δ*pslBCD*/p*_lac_-yedQ* to H_2_O_2_. (*b*) Psl was more influential than Pel in conferring ROS resistance. (*c*) Psl staining showed formation of small aggregates and synthesis of larger amounts of Psl in PAO1 treated with 0.5, 1 and 2 mM H_2_O_2_ compared with control PAO1 cultures. (*d*) Psl staining revealed synthesis of larger amounts of Psl in representative RSCVs and strains with high intracellular c-di-GMP content than PAO1. (*e*) Addition of l-arabinose to PAO1/p*_BAD_-psl* increased biofilm formation in a dose-dependent manner. (*f*) Resistance of PAO1/p*_BAD_-psl* to H_2_O_2_ increased with increasing l-arabinose concentrations. (*g*) Cellulase treatment abolished ROS resistance in PAO1/p*_BAD_-psl*. Means ± s.d. from triplicate experiments are shown; *p* < 0.05, one-way ANOVA.

The Δ*wspF* and PAO1/p*_lac_-yedQ* mutants were more resistant to 4 mM H_2_O_2_ (1× MIC) than PAO1, while PAO1/p*_lac_-yhjH* was more sensitive to 4 mM H_2_O_2_ than PAO1 (figures 4*c* and 5*a*). Epistatic mutations of *wspR* gene restored p*_cdrA_-gfp* expression (figure 4*d*) to that of the wild-type of a few selected RSCV isolates. This also caused the RSCV isolates to lose their resistance to H_2_O_2_ (figure 4*e*), confirming that PAO1 could adapt to ROS stress simply via the mutagenesis of *wspF*.

As clinical RSCV isolates with *wspF* mutations were isolated from CF patients [27], we also tested the ROS resistance of clinical RSCV isolates with known *wspF* mutations (CF173-2005 isolate of lineage H and a related isolate CF273-2002) [45]. Such RSCV isolates from the CF lungs possess higher c-di-GMP levels [8]. We showed that they were resistant to 4 mM H_2_O_2_ and the insertion of the p*_lac_-yhjH* plasmid reduced their resistance to ROS (figure 4*f*). This confirmed that the clinical RSCV isolates were adapted to the presence of ROS in the human body and cemented the involvement of c-di-GMP regulation in biofilm ROS resistance.

### 3.5. C-di-GMP-dependent exopolysaccharides are required for *Pseudomonas aeruginosa* ROS resistance

To understand how the *wsp* operon mediates ROS resistance, we tested whether exopolysaccharides could interfere with the action of ROS on *P. aeruginosa*, as the *wsp* operon regulates the expression of Pel and Psl exopolysaccharide synthetic genes [8]. We showed that both Δ*wspF*Δ*pelA*Δ*pslBCD* and Δ*pelA*Δ*pslBCD*/p*_lac_-yedQ* mutants, which could not produce Pel and Psl exopolysaccharides, were highly sensitive to H_2_O_2_, even though they contain high intracellular c-di-GMP levels (figure 5*a*). To identify which of the two exopolysaccharides were more important in conferring ROS resistance, we further showed that the Δ*pslBCD*/p*_lac_-yedQ* was more sensitive to ROS than Δ*pelA*/p*_lac_-yedQ*, thus providing the first indication that Psl is more important than Pel in conferring ROS resistance (figure 5*b*). We then stained the Psl using a fluorescent lectin and observed the formation of small aggregates and enhanced production of Psl in PAO1 with 0.5, 1 and 2 mM H_2_O_2_ treatment (figure 5*c*). Moreover, the evolved RSCVs, Δ*wspF* and PAO1/p*_lac_-yedQ* mutants produced higher amounts of Psl than PAO1, as indicated by the fluorescence intensity of the Psl stain (figure 5*d*).

We further validated the requirement of Psl for ROS resistance by using a PAO1/p*_BAD_-psl* mutant by replacing its native *psl* operon promoter with an l-arabinose-inducible promoter [58]. Upon addition of l-arabinose, there was an increase in biofilm formation via Psl production (figure 5*e*). Resistance of PAO1/p*_BAD_-psl* to ROS improved with increasing l-arabinose concentrations (figure 5*f*), indicating that Psl production is positively correlated with ROS resistance. Treatment with cellulase, which degrades Psl [58], rendered the PAO1/p*_BAD_-psl* sensitive to ROS treatment (figure 5*g*). Hence, Psl plays a major role in *P. aeruginosa* ROS resistance. Moreover, Psl acts as a signal for biofilm formation [43], thus could possibly provide positive feedback for biofilm formation and the emergence of RSCVs upon ROS exposure.

### 3.6. C-di-GMP signalling confers resistance to macrophage phagocytosis

**Figure 6.**
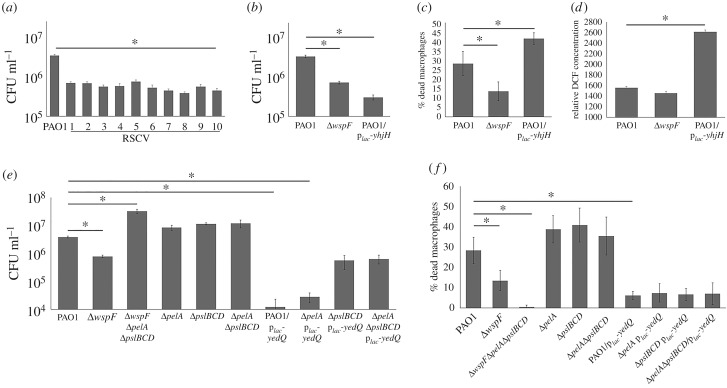
Adaptation to H_2_O_2_ provides further benefits by conferring protection against macrophages. (*a*) Quantification of phagocytosed PAO1 and evolved RSCV isolates by macrophages. (*b*) Quantification of phagocytosed PAO1, Δ*wspF* and PAO1/p*_lac_-yhjH* strains by macrophages. (*c*) Cytotoxicity assay of macrophages by PAO1, Δ*wspF* and PAO1/p*_lac_-yhjH* strains. (*d*) Quantification of ROS produced by macrophages in the presence of PAO1, Δ*wspF*, PAO1/p*_lac_-yedQ* and PAO1/p*_lac_-yhjH* strains using ROS detection assay. DCF (*e*) Quantification of phagocytosed PAO1 and Δ*pslBCD* strains by macrophages. (*f*) Cytotoxicity assay of macrophages by PAO1 and Δ*pslBCD* strains. Means ± s.d. from triplicate experiments are shown; *p* < 0.05, one-way ANOVA.

ROS are mostly produced by host leucocytes, such as PMNs and macrophages, to kill the invading pathogens, which in turn induce c-di-GMP content to confer ROS resistance. Because the evolved RSCVs and the Δ*wspF* mutant showed increased resistance to H_2_O_2_ *in vitro*, we examined whether c-di-GMP signalling could protect *P. aeruginosa* from phagocytosis by RAW264.7 macrophages. Here, the Δ*wspF* mutant and evolved RSCVs were more efficient in evading phagocytosis by macrophages than PAO1 (figure 6*a*,*b*). Although the PAO1/p*_lac_-yhjH* strain was subjected to lower phagocytosis levels by the macrophages than Δ*wspF* mutant (figure 6*b*), we found that it was highly cytotoxic to macrophages compared with PAO1 and the Δ*wspF* mutant (figure 6*c*). Hence, the lower macrophage phagocytosis level could be attributed to increased macrophage killing by PAO1/p*_lac_-yhjH*. Moreover, the PAO1/p*_lac_-yhjH* was exposed to higher ROS production from macrophages than PAO1 and the Δ*wspF* mutant (figure 6*d*).

Further, Psl played a role in evading phagocytosis by macrophages, as Δ*pslBCD*/p*_lac_-yedQ* was internalized at a higher rate than PAO1/p*_lac_-yedQ* and Δ*pelA*/p*_lac_-yedQ* (figure 6*e*). However, Psl was not involved in the cytotoxicity against macrophages, as Psl was not a virulence factor and there were no significant differences between PAO1/p*_lac_-yedQ* and Δ*pslBCD*/p*_lac_-yedQ* in killing the macrophage cells (figure 6*f*). Nonetheless, increases in c-di-GMP levels by p*_lac_-yedQ* plasmid insertion reduced the cytotoxicity to macrophages, which corroborated the Δ*wspF* mutant results (figure 6*f*).

## 4. Discussion

**Figure 7.**
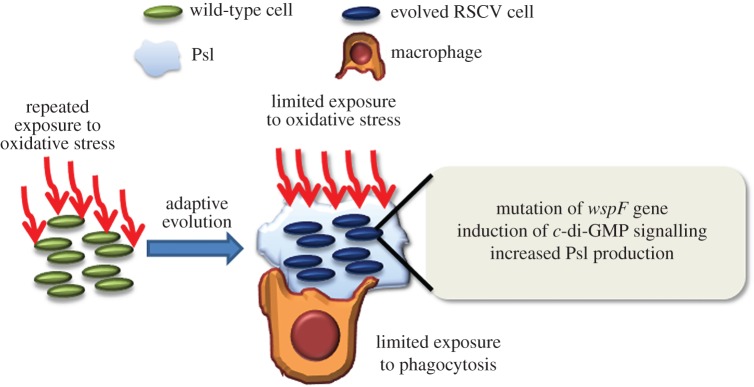
Model of *Pseudomonas aeruginosa* adaptation to oxidative stress. Upon repeated exposure of oxidative stress, the cells evolved to form RSCVs via *wspF* mutation, resulting in the induction of c-di-GMP signalling and increased Psl production. This endowed RSCVs with the ability to resist oxidative stress and phagocytes.

Resistance to oxidative stress is essential for pathogens to survive in the host environment. Here, we employed the adaptive experimental evolution assay to investigate *P. aeruginosa'*s ROS resistance via the emergence of RSCVs with enhanced biofilm forming capacity (figure 7). The production of superoxide in the phagosome was estimated at 1–4 M [59,60], implying that the use of a millimolar range of H_2_O_2_ to induce pathogen evolution in our study was relevant to the host environment. We observed an adaptive evolution trait of mutagenesis in the *wspF* gene, resulting in c-di-GMP signal induction. Our findings are clinically significant as RSCVs with *wspF* mutations are frequently isolated from CF patients and these showed similar resistance to oxidative stress as our evolved RSCVs.

The regulatory role of c-di-GMP signalling on oxidative stress response is extendable to other pathogenic species, including *Burkholderia cenocepacia* and *Klebsiella pneumoniae* (electronic supplementary material, figure S3).The ROS-induced evolution of RSCVs could potentially explain the emergence of RSCVs by *B. cenocepacia* and *K. pneumoniae* during infections [61,62]. The induction of c-di-GMP signalling has been linked to increased production of Psl exopolysaccharide in *P. aeruginosa* [50]. While Psl stiffens the biofilm structure, act as signal for biofilm formation and offers protection against antibiotics [43,63], the role of Psl as a barrier in the biofilm possibly reduces ROS penetration across bacterial membranes and causes cell damage. This confers bacterial cells with a biofilm-based protective mechanism from host immune cell generated ROS and/or antimicrobials.

As the oxidative stress resistance mechanism is commonly utilized by various pathogens, we propose the following strategies to modulate c-di-GMP signalling and treat biofilm-associated infections: (i) employing matrix-degrading enzymes, such as cellulase or the PslG hydrolase [64] to degrade Psl-like polysaccharides followed by effective killing of biofilm cells by antibiotics; and (ii) administering antioxidant drugs during early infection stages to prevent pathogen adaptation. To nullify *P. aeruginosa'*s oxidative stress adaptation in our evolution assays, we used the antioxidant l-glutathione, which reduced the emergence of RSCVs. This also indirectly reduced the resistance of *P. aeruginosa* to host immune clearance strategies such as phagocytosis. Given the lower levels of glutathione and increased oxidative stress from neutrophils in CF patients' lungs, antioxidant therapy could potentially prove effective against inflammation [65,66].

In summary, the interactions between the immune system and pathogens are highly complex, driving the pathogen to adapt accordingly to different host immune system stimuli. *Pseudomonas aeruginosa* appears to draw a complex yet fine balance between its numerous DGCs and PDEs, thus leading to phenotypic variation, and ensuring the survival of its species. Hence, chemical manipulation of the c-di-GMP signalling pathway represents a promising strategy to manage chronic bacterial infections.

## Supplementary Material

Supplementary Tables S1, S2 and S3

## Data accessibility

The Whole Genome Shotgun bioproject for *P. aeruginosa* adaptation to ROS has been deposited at in the NCBI Short Read Archive (SRA) database with accession code SRP062804.
