# Correction to ‘Reactive oxygen species drive evolution of pro-biofilm variants in pathogens by modulating cyclic-di-GMP levels’

**DOI:** 10.1098/rsob.170197

**Published:** 2017-09-20

**Authors:** Song Lin Chua, Yichen Ding, Yang Liu, Zhao Cai, Jianuan Zhou, Peter I. Benke, Sanjay Swarup, Daniela I. Drautz-Moses, Stephan Christoph Schuster, Staffan Kjelleberg, Michael Givskov, Liang Yang

*Open Biol*. 6, 160162. (Published online 23 November 2016). (doi:10.1098/rsob.160162)

A correction is required to the author list and contributions statement for ‘Reactive oxygen species drive evolution of pro-biofilm variants in pathogens by modulating cyclic-di-GMP levels’ doi:10.1098/rsob.160162. All of our authors consent to the addition of the author, Dr Peter Benke. The updated list is as follows:

Song Lin Chua^1,2^*, Yichen Ding^2,3^, Yang Liu^2^, Zhao Cai^2,3^, Jianuan Zhou^2,4^, Peter I. Benke^2,6^, Sanjay Swarup^2,5,6^, Daniela I. Drautz-Moses^2^, Stephan Christoph Schuster^2,7^, Staffan Kjelleberg^2,6,8^, Michael Givskov^2,9^, Liang Yang^2,6^*

^1^Lee Kong Chian School of Medicine, Nanyang Technological University, Singapore 639798

^2^Singapore Centre for Environmental Life Sciences Engineering (SCELSE), Nanyang Technological University, Singapore 637551

^3^Interdisciplinary Graduate School, Nanyang Technological University, Singapore 637551

^4^Integrative Microbiology Research Centre, South China Agricultural University, Guangzhou, 510642, People's Republic of China.

^5^Department of Biological Sciences, National University of Singapore, Singapore 117543

^6^NUS Environmental Research Institute, National University of Singapore, Singapore

^7^School of Biological Sciences, Nanyang Technological University, Singapore 639798

^8^Center for Marine Bio-Innovation and School of Biotechnology and Biomolecular Sciences, University of New South Wales, Sydney 2052, Australia

^9^Costerton Biofilm Center, Department of Immunology and Microbiology, University of Copenhagen, 2200 København N, Denmark

## Corrected Authors' contributions

S.L.C., S.K., M.G. and L.Y. designed the research. S.L.C., Y.D. and J.Z. performed the experiments. Z.C. conducted the extraction of c-di-GMP from samples, normalization of c-di-GMP levels with protein and interpretation of results; P.B. conducted LC-MS experiment for c-di-GMP; S.S. discussed the experiment of c-di-GMP quantification of samples and interpretation of results with relevance to project. Y.L., D.I.D. and S.C.S. performed and analysed DNA sequencing experiments. S.K. and M.G. analysed the data. S.L.C. and L.Y. wrote the paper. All authors read and approved the final manuscript.

