# Supplementary material for: Reactive oxygen species drive evolution of pro-biofilm variants in pathogens by modulating cyclic-di-GMP levels
Source: Open Biol. 2016 Nov 23;6(11):160162. doi: 10.1098/rsob.160162 (PMC5133437; doi:10.1098/rsob.160162)
Supplement: Supplementary Tables S1, S2 and S3 [file rsob160162supp1.pdf]

Supplementary Table S1: Bacterial strains and plasmids

Strain or plasmid	Relevant characteristics	Source or reference
P. aeruginosa		
PAO1	Prototypic non-mucoid wild-type strain	[1]
PAO1/Δ*wspF*	*wspF* knockout of PAO1 constructed by allelic exchange and sequenced	[2]
PAO1/Δ*wspF*Δ*pelA*Δ*pslBCD*	*wspF*, *pelA*, *pslBCD* triple knockout of PAO1 constructed by allelic exchange	[3]
PAO1/Δ*wspR*	*wspR* knockout of PAO1 constructed by allelic exchange	This study
PAO1/p_{BAD}-*psl*	PAO1 with *psl* gene with arabinose-inducible *BAD* promoter	[4]

PAO1/p_{lac}-*yhjH*	Tc^r; PAO1 containing the p_{lac}-*yhjH* vector	[5]
PAO1/Δ*pelA*	*pelA* knockout of PAO1 constructed by allelic exchange	[6]
PAO1/Δ*pslBCD*	*pslBCD* knockout of PAO1 constructed by allelic exchange	[6]
PAO1/Δ*pelA*Δ*pslBCD*	*pelA* and *pslBCD* double knockout of PAO1 constructed by allelic exchange	[6]
PAO1/p_{lac}-*yedQ*	Gm^r; PAO1 containing the p_{lac}-*yedQ* vector	[3]
PAO1/Δ*pelA*/p_{lac}-*yedQ*	Gm^r; PAO1/Δ*pelA* containing the p_{lac}-*yedQ* vector	[3]
PAO1/Δ*pslBCD*/p_{lac}-*yedQ*	Gm^r; PAO1/Δ*pslBCD* containing the p_{lac}-*yedQ*	[3]

	vector	
PAO1/Δ*pelA*Δ*pslBCD*/p$_{lac}$-*yedQ*	Gm^r; PAO1/Δ*pelA*Δ*pslBCD* containing the p$_{lac}$-*yedQ* vector	[3]
PAO1/p$_{cdrA}$-*gfp*	Gm^r; PAO1 containing the p$_{cdrA}$-*gfp* vector	[2]
B. cenocepacia		
B. cenocepacia 111	Prototypic wild-type strain	[7]
B. cenocepacia/p$_{lac}$-*yedQ*	Gm^r; *B. cenocepacia* 111 containing the p$_{lac}$-*yedQ* vector	This study
B. cenocepacia/ p$_{lac}$-*yhjH*	Tc^r; *B. cenocepacia* 111 containing the p$_{lac}$-*yhjH* vector	This study
K. pneumoniae		
K. pneumoniae KP-1	Prototypic wild-type strain	[8]
K. pneumoniae/p$_{lac}$-*yedQ*	Gm^r; *K. pneumoniae* containing the p$_{lac}$-*yedQ*	This study

	vector	
K. pneumoniael p_{lac}-*yhjH*	Tc^r ; *K. pneumoniae* containing the p_{lac}-*yhjH* vector	This study
E. coli		
DH5α	F–, ø80dlacZΔM15, Δ(lacZYAargF)U169, deoR, recA1, endA1, hsdR17(rK–, mK+), phoA, supE44, λ–, thi-1,gyrA96, relA1	Laboratory Collection
**Plasmids**		
pUCp22	Ap^r Gm^r; broad-host-range cloning vector	[9]
pRK600	Cm^r; ori ColE1 $RK2^-Mob^+$ $RK2^-Tra^+$; helper vector for conjugation	[10]
p_{lac}-*yedQ*	Gm^r; pUCP22 carrying the *yedQ* gene	[3]

p*lac*-*yhjH*	Tc^r; pBBR1MCS3 carrying the *yhjH* gene	5
p*cdrA*-*gfp*	Apr Gm^r; pUCP22 carrying the p*cdrA*-*gfp* fusion	2

Table S2. Summary of genes/ regions that were mutated in the PAO1 control colonies and RSCV isolates compared to the PAO1 ancestor.

Control 1

Region/ Gene with mutations	Functional Group
64793	Non-coding region
721718	Non-coding region
788573	Non-coding region
788581	Non-coding region
789185	Non-coding region
4699910	Non-coding region
5242141	Non-coding region
PA0720	helix destabilizing protein of bacteriophage Pf1
PA1458	two-component sensor
exsC	exoenzyme S synthesis protein C
PA2877	transcriptional regulator

Control 2

Region/ Gene with mutations	Functional Group
64793	Non-coding region
721622	Non-coding region

788573	Non-coding region
788581	Non-coding region
789170	Non-coding region
789185	Non-coding region
794290	Non-coding region
794291	Non-coding region
4699910	Non-coding region
5242141	Non-coding region
PA0727	Hypothetical protein
PA0720	helix destabilizing protein of bacteriophage Pf1
PA1458	two-component sensor
exsC	exoenzyme S synthesis protein C
PA2877	transcriptional regulator

RSCV 1

Region/ Gene with mutations	Functional Group
64793	Non-coding region
721622	Non-coding region
788573	Non-coding region
788581	Non-coding region
789170	Non-coding region
789185	Non-coding region
794290	Non-coding region
794291	Non-coding region
4699910	Non-coding region
5242141	Non-coding region
PA0727	Hypothetical protein
PA0720	helix destabilizing protein of bacteriophage Pf1
PA1458	two-component sensor
exsC	exoenzyme S synthesis protein C

PA2877	transcriptional regulator
wspF	chemotaxis-specific methylesterase
mexT	transcriptional regulator MexT

RSCV 2

Region/ Gene with mutations	Functional Group
64793	Non-coding region
721670	Non-coding region
721718	Non-coding region
721725	Non-coding region
721740	Non-coding region
788573	Non-coding region
788581	Non-coding region
789170	Non-coding region
789185	Non-coding region
789266	Non-coding region
789332	Non-coding region
4699910	Non-coding region
5242141	Non-coding region
PA0726	Hypothetical protein
PA0727	Hypothetical protein
PA2046	Hypothetical protein
PA0720	helix destabilizing protein of bacteriophage Pf1
exsC	exoenzyme S synthesis protein C
PA2877	transcriptional regulator
wspF	chemotaxis-specific methylesterase

RSCV 3

Region/ Gene with mutations	Functional Group
64793	Non-coding region

721611	Non-coding region
788573	Non-coding region
788581	Non-coding region
789170	Non-coding region
789185	Non-coding region
789266	Non-coding region
789288	Non-coding region
790604	Non-coding region
4699910	Non-coding region
5242141	Non-coding region
PA0718	Hypothetical protein
PA2451	Hypothetical protein
PA0721	Hypothetical protein
PA0725	Hypothetical protein
PA0727	Hypothetical protein
PA0683	type II secretion system protein
PA0720	helix destabilizing protein of bacteriophage Pf1
PA0724	phage coat protein A
PA1458	two-component sensor
exsC	exoenzyme S synthesis protein C
PA2877	transcriptional regulator
wspF	chemotaxis-specific methylesterase

RSCV 4

Region/ Gene with mutations	Functional Group
64793	Non-coding region
721670	Non-coding region
721718	Non-coding region
721725	Non-coding region
721740	Non-coding region

788573	Non-coding region
788581	Non-coding region
789170	Non-coding region
789185	Non-coding region
789266	Non-coding region
4699910	Non-coding region
5242141	Non-coding region
PA0721	hypothetical protein
PA0727	Hypothetical protein
PA0724	phage coat protein A
PA0720	helix destabilizing protein of bacteriophage Pf1
PA1458	two-component sensor
exsC	exoenzyme S synthesis protein C
PA2877	transcriptional regulator
wspF	chemotaxis-specific methylesterase
fha1	Fha domain-containing protein

RSCV 5

Region/ Gene with mutations	Functional Group
64793	Non-coding region
721670	Non-coding region
721718	Non-coding region
721725	Non-coding region
721740	Non-coding region
788573	Non-coding region
788581	Non-coding region
789170	Non-coding region
789185	Non-coding region
789266	Non-coding region
794430	Non-coding region

PA0726	hypothetical protein
PA0724	phage coat protein A
PA1458	two-component sensor
exsC	exoenzyme S synthesis protein C
PA2877	transcriptional regulator
wspF	chemotaxis-specific methylesterase
fha1	Fha domain-containing protein

RSCV 6

Region/ Gene with mutations	Functional Group
64793	Non-coding region
721670	Non-coding region
788573	Non-coding region
788581	Non-coding region
789170	Non-coding region
789185	Non-coding region
789266	Non-coding region
789288	Non-coding region
PA0727	hypothetical protein
PA0748	still frameshift probable transcriptional regulator
PA0724	phage coat protein A
napA	nitrate reductase catalytic subunit
PA1458	two-component sensor
exsC	exoenzyme S synthesis protein C
PA2877	transcriptional regulator
wspF	chemotaxis-specific methylesterase
uvrD	DNA-dependent helicase II

RSCV 7

Region/ Gene with mutations	Functional Group

64793	Non-coding region
721670	Non-coding region
721718	Non-coding region
721725	Non-coding region
721740	Non-coding region
788573	Non-coding region
788581	Non-coding region
789170	Non-coding region
789185	Non-coding region
4699910	Non-coding region
PA0727	hypothetical protein
exsC	exoenzyme S synthesis protein C
PA2877	transcriptional regulator
wspF	chemotaxis-specific methylesterase

RSCV 8

Region/ Gene with mutations	Functional Group
64793	Non-coding region
721670	Non-coding region
721725	Non-coding region
788573	Non-coding region
788581	Non-coding region
789170	Non-coding region
789185	Non-coding region
789266	Non-coding region
789288	Non-coding region
789332	Non-coding region
4699910	Non-coding region
5242141	Non-coding region
PA0718	hypothetical protein

PA0727	hypothetical protein
PA1458	two-component sensor
exsC	exoenzyme S synthesis protein C
PA2877	transcriptional regulator
wspF	chemotaxis-specific methylesterase

RSCV 9

Region/ Gene with mutations	Functional Group
64793	Non-coding region
721670	Non-coding region
721718	Non-coding region
721725	Non-coding region
721740	Non-coding region
788573	Non-coding region
788581	Non-coding region
789170	Non-coding region
789185	Non-coding region
789266	Non-coding region
789288	Non-coding region
789332	Non-coding region
794430	Non-coding region
4699910	Non-coding region
5242141	Non-coding region
PA0718	hypothetical protein
PA0726	hypothetical protein
PA0727	hypothetical protein
PA1458	two-component sensor
exsC	exoenzyme S synthesis protein C
PA2877	transcriptional regulator
wspF	chemotaxis-specific methylesterase

RSCV 10

Region/ Gene with mutations	Functional Group
64793	Non-coding region
64793	Non-coding region
721670	Non-coding region
721718	Non-coding region
721725	Non-coding region
721740	Non-coding region
788573	Non-coding region
788581	Non-coding region
789170	Non-coding region
789185	Non-coding region
789266	Non-coding region
789288	Non-coding region
789332	Non-coding region
4699910	Non-coding region
PA0718	hypothetical protein
PA0727	hypothetical protein
PA1458	two-component sensor
exsC	exoenzyme S synthesis protein C
PA2877	transcriptional regulator
wspF	chemotaxis-specific methylesterase

Table S3. Mutations identified in the PAO1 control colonies and RSCV isolates compared to the PAO1 ancestor.

Control 1

Region	Type	Gene	Original sequence	Sequence Change	Amino acid change	Functional Group

64793	SNV		A	A		
64793	SNV		A	G		
721718	SNV		A	G		
788573	SNV		T	C		
788573	SNV		T	T		
788581	SNV		G	G		
788581	SNV		G	T		
789185	SNV		A	A		
789185	SNV		A	C		
790219	SNV	PA0720	C	C		helix destabilizing protein of bacteriophage Pf1
790219	SNV	PA0720	C	T		helix destabilizing protein of bacteriophage Pf1
794612	SNV	PA0727	C	C		hypothetical protein
794612	SNV	PA0727	C	T		hypothetical protein
794650	SNV	PA0727	G	C		hypothetical protein
794650	SNV	PA0727	G	G		hypothetical protein
795025	SNV	PA0727	G	C		hypothetical protein
795025	SNV	PA0727	G	G		hypothetical protein
795028	SNV	PA0727	G	G		hypothetical protein
795028	SNV	PA0727	G	T		hypothetical protein
795304	SNV	PA0727	C	C		hypothetical protein
795304	SNV	PA0727	C	T		hypothetical protein
795385	SNV	PA0727	T	C		hypothetical protein
795385	SNV	PA0727	T	T		hypothetical protein
795391	SNV	PA0727	G	C		hypothetical protein
795391	SNV	PA0727	G	G		hypothetical protein
795403	SNV	PA0727	C	C		hypothetical protein
795403	SNV	PA0727	C	T		hypothetical protein
795406	SNV	PA0727	G	A		hypothetical protein
795406	SNV	PA0727	G	G		hypothetical protein

795409	SNV	PA0727	A	A		hypothetical protein
795409	SNV	PA0727	A	G		hypothetical protein
795412	SNV	PA0727	A	A		hypothetical protein
795412	SNV	PA0727	A	G		hypothetical protein
795424	SNV	PA0727	C	C		hypothetical protein
795424	SNV	PA0727	C	T		hypothetical protein
795434	SNV	PA0727	A	A		hypothetical protein
795434	SNV	PA0727	A	G	NP_249418.1:p.Ser312Gly	hypothetical protein
795667	SNV	PA0727	T	C		hypothetical protein
795667	SNV	PA0727	T	T		hypothetical protein
795671	SNV	PA0727	C	C		hypothetical protein
795671	SNV	PA0727	C	T		hypothetical protein
795742	SNV	PA0727	C	C		hypothetical protein
795742	SNV	PA0727	C	T		hypothetical protein
795745	SNV	PA0727	A	A		hypothetical protein
795745	SNV	PA0727	A	C		hypothetical protein
795754	SNV	PA0727	G	C		hypothetical protein
795754	SNV	PA0727	G	G		hypothetical protein
795766	SNV	PA0727	G	C		hypothetical protein
795766	SNV	PA0727	G	G		hypothetical protein
1587916	SNV	PA1458	T	C		two-component sensor
1587919	SNV	PA1458	G	T	NP_250149.1:p.Glu299Asp	two-component sensor
1856056	SNV	exsC	C	C		exoenzyme S synthesis protein C
1856056	SNV	exsC	C	G		exoenzyme S synthesis protein C
1856058	SNV	exsC	A	A		exoenzyme S synthesis protein C
1856058	SNV	exsC	A	G	NP_250401.1:p.Glu66Gly	exoenzyme S synthesis protein C
3230295	SNV	PA2877	A	A		transcriptional regulator
3230295	SNV	PA2877	A	G	NP_251567.1:p.Leu293Pro	transcriptional regulator
4699910	Deletion		C	-		
5242141	SNV		G	C		

5242141	SNV		G	G		
721663..721664	MNV		TC	CT		
794989..794990	MNV	PA0727	CT	CT		hypothetical protein
794989..794990	MNV	PA0727	CT	GC		hypothetical protein
795009..795010	MNV	PA0727	AA	AA		hypothetical protein
795009..795010	MNV	PA0727	AA	CG	NP_249418.1:p.Lys170Thr	hypothetical protein
795019^795020	Insertion	PA0727	-	-		hypothetical protein
795019^795020	Insertion	PA0727	-	T	NP_249418.1:p.Asp174fs	hypothetical protein

Control 2

Region	Type	Gene	Original sequence	Sequence Change	Amino acid change	Functional Group
64793	SNV		A	A		
64793	SNV		A	G		
721622	SNV		C	T		
788573	SNV		T	C		
788573	SNV		T	T		
788581	SNV		G	G		
788581	SNV		G	T		
789170	SNV		C	C		
789170	SNV		C	T		
789185	SNV		A	A		
789185	SNV		A	C		
794290	SNV		C	C		

794290	SNV		C	T		
794291	SNV		C	T		
795025	SNV	PA0727	G	C		hypothetical protein
795025	SNV	PA0727	G	G		hypothetical protein
795028	SNV	PA0727	G	G		hypothetical protein
795028	SNV	PA0727	G	T		hypothetical protein
795301	SNV	PA0727	A	A		hypothetical protein
795301	SNV	PA0727	A	G		hypothetical protein
795310	SNV	PA0727	C	C		hypothetical protein
795310	SNV	PA0727	C	T		hypothetical protein
795313	SNV	PA0727	T	C		hypothetical protein
795313	SNV	PA0727	T	T		hypothetical protein
795349	SNV	PA0727	C	C		hypothetical protein
795349	SNV	PA0727	C	T		hypothetical protein
795355	SNV	PA0727	T	C		hypothetical protein
795355	SNV	PA0727	T	T		hypothetical protein
795370	SNV	PA0727	T	C		hypothetical protein
795370	SNV	PA0727	T	T		hypothetical protein
795391	SNV	PA0727	G	C		hypothetical protein
795391	SNV	PA0727	G	G		hypothetical protein
795406	SNV	PA0727	G	A		hypothetical protein
795406	SNV	PA0727	G	G		hypothetical protein
795409	SNV	PA0727	A	A		hypothetical protein
795409	SNV	PA0727	A	G		hypothetical protein
795412	SNV	PA0727	A	A		hypothetical protein
795412	SNV	PA0727	A	G		hypothetical protein
795424	SNV	PA0727	C	C		hypothetical protein
795424	SNV	PA0727	C	T		hypothetical protein
795667	SNV	PA0727	T	C		hypothetical protein
795667	SNV	PA0727	T	T		hypothetical protein

795671	SNV	PA0727	C	C		hypothetical protein	
795671	SNV	PA0727	C	T		hypothetical protein	
795745	SNV	PA0727	A	A		hypothetical protein	
795745	SNV	PA0727	A	C		hypothetical protein	
795754	SNV	PA0727	G	C		hypothetical protein	
795754	SNV	PA0727	G	G		hypothetical protein	
795766	SNV	PA0727	G	C		hypothetical protein	
795766	SNV	PA0727	G	G		hypothetical protein	
1587916	SNV	PA1458	T	C		two-component sensor	
1587919	SNV	PA1458	G	T	NP_250149.1:p.Glu299Asp	two-component sensor	
1587941	SNV	PA1458	T	G	NP_250149.1:p.Ser307Ala	two-component sensor	
1587943	SNV	PA1458	G	A		two-component sensor	
1856056	SNV	exsC	C	C		exoenzyme S synthesis protein C	
1856056	SNV	exsC	C	G		exoenzyme S synthesis protein C	
1856058	SNV	exsC	A	A		exoenzyme S synthesis protein C	
1856058	SNV	exsC	A	G	NP_250401.1:p.Glu66Gly	exoenzyme S synthesis protein C	
3230295	SNV	PA2877	A	A		transcriptional regulator	
3230295	SNV	PA2877	A	G	NP_251567.1:p.Leu293Pro	transcriptional regulator	
4699910	Deletion		C	-			
5242141	SNV		G	C			
5242141	SNV		G	G			
789219..789220	MNV		GG	AT			
789219..789220	MNV		GG	GG			
794290..794291	MNV		CC	CC			
794290..794291	MNV		CC	TT			
794989..794990	MNV	PA0727	CT	CT		hypothetical protein	
794989..794990	MNV	PA0727	CT	GC		hypothetical protein	
795009..795010	MNV	PA0727	AA	AA		hypothetical protein	
795009..795010	MNV	PA0727	AA	CG	NP_249418.1:p.Lys170Thr	hypothetical protein	
795019^795020	Insertion	PA0727	-	-		hypothetical protein	

795019^795020	Insertion	PA0727	-	T	NP_249418.1:p.Asp174fs	hypothetical protein

RSCV 1

Region	Type	Gene	Original sequence	Sequence Change	Amino acid change	Functional Group
64793	SNV		A	A		
64793	SNV		A	G		
721622	SNV		C	T		
788573	SNV		T	C		
788573	SNV		T	T		
788581	SNV		G	G		
788581	SNV		G	T		
789170	SNV		C	C		
789170	SNV		C	T		
789185	SNV		A	A		
789185	SNV		A	C		
794290	SNV		C	C		

	794290	SNV		C	T			
	794291	SNV		C	T			
	795025	SNV	PA0727	G	C		hypothetical protein	
	795025	SNV	PA0727	G	G		hypothetical protein	
	795028	SNV	PA0727	G	G		hypothetical protein	
	795028	SNV	PA0727	G	T		hypothetical protein	
	795301	SNV	PA0727	A	A		hypothetical protein	
	795301	SNV	PA0727	A	G		hypothetical protein	
	795310	SNV	PA0727	C	C		hypothetical protein	
	795310	SNV	PA0727	C	T		hypothetical protein	
	795313	SNV	PA0727	T	C		hypothetical protein	
	795313	SNV	PA0727	T	T		hypothetical protein	
	795349	SNV	PA0727	C	C		hypothetical protein	
	795349	SNV	PA0727	C	T		hypothetical protein	
	795355	SNV	PA0727	T	C		hypothetical protein	
	795355	SNV	PA0727	T	T		hypothetical protein	
	795370	SNV	PA0727	T	C		hypothetical protein	
	795370	SNV	PA0727	T	T		hypothetical protein	
	795391	SNV	PA0727	G	C		hypothetical protein	
	795391	SNV	PA0727	G	G		hypothetical protein	
	795406	SNV	PA0727	G	A		hypothetical protein	
	795406	SNV	PA0727	G	G		hypothetical protein	
	795409	SNV	PA0727	A	A		hypothetical protein	
	795409	SNV	PA0727	A	G		hypothetical protein	
	795412	SNV	PA0727	A	A		hypothetical protein	
	795412	SNV	PA0727	A	G		hypothetical protein	
	795424	SNV	PA0727	C	C		hypothetical protein	
	795424	SNV	PA0727	C	T		hypothetical protein	
	795667	SNV	PA0727	T	C		hypothetical protein	
	795667	SNV	PA0727	T	T		hypothetical protein	

795671	SNV	PA0727	C	C		hypothetical protein
795671	SNV	PA0727	C	T		hypothetical protein
795745	SNV	PA0727	A	A		hypothetical protein
795745	SNV	PA0727	A	C		hypothetical protein
795754	SNV	PA0727	G	C		hypothetical protein
795754	SNV	PA0727	G	G		hypothetical protein
795766	SNV	PA0727	G	C		hypothetical protein
795766	SNV	PA0727	G	G		hypothetical protein
1587916	SNV	PA1458	T	C		two-component sensor
1587919	SNV	PA1458	G	T	NP_250149.1:p.Glu299Asp	two-component sensor
1587941	SNV	PA1458	T	G	NP_250149.1:p.Ser307Ala	two-component sensor
1587943	SNV	PA1458	G	A		two-component sensor
1856056	SNV	exsC	C	C		exoenzyme S synthesis protein C
1856056	SNV	exsC	C	G		exoenzyme S synthesis protein C
1856058	SNV	exsC	A	A		exoenzyme S synthesis protein C
1856058	SNV	exsC	A	G	NP_250401.1:p.Glu66Gly	exoenzyme S synthesis protein C
3230295	SNV	PA2877	A	A		transcriptional regulator
3230295	SNV	PA2877	A	G	NP_251567.1:p.Leu293Pro	transcriptional regulator
4145193	SNV	wspF	A	G	NP_252392.1:p.Ser252Pro	chemotaxis-specific methylesterase
4699910	Deletion		C	-		
5242141	SNV		G	C		
5242141	SNV		G	G		
2807694..2807701	Deletion	mexT	CGGCCAGC	-	NP_251182.1:p.Gln80fs	transcriptional regulator MexT
2807694..2807701	MNV	mexT	CGGCCAGC	CGGCCAGC		transcriptional regulator MexT
789219..789220	MNV		GG	AT		
789219..789220	MNV		GG	GG		
794290..794291	MNV		CC	CC		
794290..794291	MNV		CC	TT		
794989..794990	MNV	PA0727	CT	CT		hypothetical protein

794989..794990	MNV	PA0727	CT	GC		hypothetical protein
795009..795010	MNV	PA0727	AA	AA		hypothetical protein
795009..795010	MNV	PA0727	AA	CG	NP_249418.1:p.Lys170Thr	hypothetical protein
795019^795020	Insertion	PA0727	-	-		hypothetical protein
795019^795020	Insertion	PA0727	-	T	NP_249418.1:p.Asp174fs	hypothetical protein

RSCV 2

Region	Type	Gene	Original sequence	Sequence Change	Amino acid change	Functional Group
64793	SNV		A	A		
64793	SNV		A	G		
721670	SNV		A	G		
721718	SNV		A	G		
721725	SNV		C	T		
721740	SNV		C	T		
788573	SNV		T	C		
788573	SNV		T	T		
788581	SNV		G	G		
788581	SNV		G	T		
789170	SNV		C	C		
789170	SNV		C	T		

789185	SNV		A	A		
789185	SNV		A	C		
789266	SNV		C	C		
789266	SNV		C	T		
789332	SNV		C	C		
789332	SNV		C	T		
790485	SNV	PA0720	T	C	NP_249411.1:p.Met107Thr	helix destabilizing protein of bacteriophage Pf1
790486	SNV	PA0720	G	C	NP_249411.1:p.Met107Ile	helix destabilizing protein of bacteriophage Pf1
790486	SNV	PA0720	G	G		helix destabilizing protein of bacteriophage Pf1
793242	SNV	PA0726	T	G		hypothetical protein
793242	SNV	PA0726	T	T		hypothetical protein
795025	SNV	PA0727	G	C		hypothetical protein
795025	SNV	PA0727	G	G		hypothetical protein
795028	SNV	PA0727	G	G		hypothetical protein
795028	SNV	PA0727	G	T		hypothetical protein
795296	SNV	PA0727	C	A	NP_249418.1:p.Leu266Ile	hypothetical protein
795296	SNV	PA0727	C	C		hypothetical protein
795310	SNV	PA0727	C	C		hypothetical protein
795310	SNV	PA0727	C	T		hypothetical protein
795313	SNV	PA0727	T	C		hypothetical protein
795313	SNV	PA0727	T	T		hypothetical protein
795349	SNV	PA0727	C	C		hypothetical protein
795349	SNV	PA0727	C	T		hypothetical protein
795385	SNV	PA0727	T	C		hypothetical protein
795385	SNV	PA0727	T	T		hypothetical protein
795391	SNV	PA0727	G	C		hypothetical protein
795391	SNV	PA0727	G	G		hypothetical protein
795403	SNV	PA0727	C	C		hypothetical protein

795403	SNV	PA0727	C	T		hypothetical protein
795409	SNV	PA0727	A	A		hypothetical protein
795409	SNV	PA0727	A	G		hypothetical protein
795412	SNV	PA0727	A	A		hypothetical protein
795412	SNV	PA0727	A	G		hypothetical protein
795424	SNV	PA0727	C	C		hypothetical protein
795424	SNV	PA0727	C	T		hypothetical protein
795432	Deletion	PA0727	A	-	NP_249418.1:p.Ser312fs	hypothetical protein
795432	SNV	PA0727	A	A		hypothetical protein
795434	SNV	PA0727	A	A		hypothetical protein
795434	SNV	PA0727	A	G	NP_249418.1:p.Ser312Gly	hypothetical protein
795472	SNV	PA0727	G	C		hypothetical protein
795472	SNV	PA0727	G	G		hypothetical protein
795667	SNV	PA0727	T	C		hypothetical protein
795667	SNV	PA0727	T	T		hypothetical protein
795671	SNV	PA0727	C	C		hypothetical protein
795671	SNV	PA0727	C	T		hypothetical protein
795742	SNV	PA0727	C	C		hypothetical protein
795742	SNV	PA0727	C	T		hypothetical protein
795745	SNV	PA0727	A	A		hypothetical protein
795745	SNV	PA0727	A	C		hypothetical protein
795754	SNV	PA0727	G	C		hypothetical protein
795754	SNV	PA0727	G	G		hypothetical protein
795766	SNV	PA0727	G	C		hypothetical protein
795766	SNV	PA0727	G	G		hypothetical protein
1856056	SNV	exsC	C	C		exoenzyme S synthesis protein C
1856056	SNV	exsC	C	G		exoenzyme S synthesis protein C
1856058	SNV	exsC	A	A		exoenzyme S synthesis protein C
1856058	SNV	exsC	A	G	NP_250401.1:p.Glu66Gly	exoenzyme S synthesis protein C

2239547	SNV	PA2046	T	G		
3230295	SNV	PA2877	A	A		transcriptional regulator
3230295	SNV	PA2877	A	G	NP_251567.1:p.Leu293Pro	transcriptional regulator
4145673	SNV	wspF	C	A	NP_252392.1:p.Glu92*	chemotaxis-specific methylesterase
4699910	Deletion		C	-		
5242141	SNV		G	C		
5242141	SNV		G	G		
2186927^2186928	Insertion		-	C		
721663..721664	MNV		TC	CT		
789219..789220	MNV		GG	AT		
789219..789220	MNV		GG	GG		
790485..790486	MNV	PA0720	TG	CC	NP_249411.1:p.Met107Thr	helix destabilizing protein of bacteriophage Pf1
790485..790486	MNV	PA0720	TG	TG		helix destabilizing protein of bacteriophage Pf1
794989..794990	MNV	PA0727	CT	CT		hypothetical protein
794989..794990	MNV	PA0727	CT	GC		hypothetical protein
795009..795010	MNV	PA0727	AA	AA		hypothetical protein
795009..795010	MNV	PA0727	AA	CG	NP_249418.1:p.Lys170Thr	hypothetical protein
795019^795020	Insertion	PA0727	-	-		hypothetical protein
795019^795020	Insertion	PA0727	-	T	NP_249418.1:p.Asp174fs	hypothetical protein

RSCV 3

Region	Type	Gene	Original sequence	Sequence Change	Amino acid change	Functional Group
64793	SNV		A	A		
64793	SNV		A	G		
721611	SNV		C	T		
788573	SNV		T	C		
788573	SNV		T	T		
788581	SNV		G	G		
788581	SNV		G	T		
789170	SNV		C	C		
789170	SNV		C	T		
789185	SNV		A	A		
789185	SNV		A	C		
789266	SNV		C	C		

789266	SNV		C	T		
789288	SNV		C	C		
789288	SNV		C	T		
789409	SNV	PA0718	C	C		hypothetical protein
789409	SNV	PA0718	C	G	NP_249409.1:p.Ala17Gly	hypothetical protein
789439	SNV	PA0718	T	C	NP_249409.1:p.Val27Ala	hypothetical protein
789439	SNV	PA0718	T	T		hypothetical protein
789470	SNV	PA0718	A	A		hypothetical protein
789470	SNV	PA0718	A	G		hypothetical protein
789473	SNV	PA0718	C	C		hypothetical protein
789473	SNV	PA0718	C	T		hypothetical protein
789476	SNV	PA0718	T	C		hypothetical protein
789476	SNV	PA0718	T	T		hypothetical protein
789505	SNV	PA0718	G	A	NP_249409.1:p.Gly49Asp	hypothetical protein
789505	SNV	PA0718	G	G		hypothetical protein
790477	SNV	PA0720	C	C		helix destabilizing protein of<br>bacteriophage<br>Pf1
790477	SNV	PA0720	C	T		helix destabilizing protein of<br>bacteriophage<br>Pf1
790485	SNV	PA0720	T	C	NP_249411.1:p.Met107Thr	helix destabilizing protein of<br>bacteriophage<br>Pf1
790485	SNV	PA0720	T	T		helix destabilizing protein of<br>bacteriophage<br>Pf1
790580	SNV	PA0720	A	A		helix destabilizing protein of<br>bacteriophage<br>Pf1
790580	SNV	PA0720	A	T	NP_249411.1:p.Thr139Ser	helix destabilizing protein of<br>bacteriophage<br>Pf1
790597	SNV	PA0720	G	C		helix destabilizing protein of<br>bacteriophage

						Pf1
790597	SNV	PA0720	G	G		helix destabilizing protein of bacteriophage Pf1
790604	SNV		C	C		
790604	SNV		C	G		
790640	SNV	PA0721	C	C		hypothetical protein
790640	SNV	PA0721	C	T		hypothetical protein
792119	SNV	PA0724	C	C		phage coat protein A
792119	SNV	PA0724	C	T		phage coat protein A
792124	SNV	PA0724	G	A	NP_249415.1:p.Gly252Asp	phage coat protein A
792124	SNV	PA0724	G	G		phage coat protein A
792359	SNV	PA0724	C	C		phage coat protein A
792359	SNV	PA0724	C	T		phage coat protein A
792401	SNV	PA0724	C	A	NP_249415.1:p.Asp344Glu	phage coat protein A
792401	SNV	PA0724	C	C		phage coat protein A
792443	SNV	PA0724	T	C		phage coat protein A
792443	SNV	PA0724	T	T		phage coat protein A
792455	SNV	PA0724	C	C		phage coat protein A
792455	SNV	PA0724	C	T		phage coat protein A
792590	SNV	PA0724	G	A		phage coat protein A
792590	SNV	PA0724	G	G		phage coat protein A
792608	SNV	PA0724	C	C		phage coat protein A
792608	SNV	PA0724	C	T		phage coat protein A
792696	SNV	PA0725	C	C		hypothetical protein
792696	SNV	PA0725	C	T		hypothetical protein
794740	SNV	PA0727	G	A		hypothetical protein
794740	SNV	PA0727	G	G		hypothetical protein
794797	SNV	PA0727	T	C		hypothetical protein
794797	SNV	PA0727	T	T		hypothetical protein

794806	SNV	PA0727	T	C		hypothetical protein
794806	SNV	PA0727	T	T		hypothetical protein
795025	SNV	PA0727	G	C		hypothetical protein
795025	SNV	PA0727	G	G		hypothetical protein
795028	SNV	PA0727	G	G		hypothetical protein
795028	SNV	PA0727	G	T		hypothetical protein
795289	SNV	PA0727	C	C		hypothetical protein
795289	SNV	PA0727	C	G		hypothetical protein
795296	SNV	PA0727	C	A	NP_249418.1:p.Leu266Ile	hypothetical protein
795296	SNV	PA0727	C	C		hypothetical protein
795301	SNV	PA0727	A	A		hypothetical protein
795301	SNV	PA0727	A	G		hypothetical protein
795304	SNV	PA0727	C	C		hypothetical protein
795304	SNV	PA0727	C	T		hypothetical protein
795310	SNV	PA0727	C	C		hypothetical protein
795310	SNV	PA0727	C	T		hypothetical protein
795313	SNV	PA0727	T	C		hypothetical protein
795313	SNV	PA0727	T	T		hypothetical protein
795349	SNV	PA0727	C	C		hypothetical protein
795349	SNV	PA0727	C	T		hypothetical protein
795355	SNV	PA0727	T	C		hypothetical protein
795355	SNV	PA0727	T	T		hypothetical protein
795385	SNV	PA0727	T	C		hypothetical protein
795385	SNV	PA0727	T	T		hypothetical protein
795391	SNV	PA0727	G	C		hypothetical protein
795391	SNV	PA0727	G	G		hypothetical protein
795403	SNV	PA0727	C	C		hypothetical protein
795403	SNV	PA0727	C	T		hypothetical protein
795406	SNV	PA0727	G	A		hypothetical protein
795406	SNV	PA0727	G	G		hypothetical protein

795409	SNV	PA0727	A	A		hypothetical protein	
795409	SNV	PA0727	A	G		hypothetical protein	
795412	SNV	PA0727	A	A		hypothetical protein	
795412	SNV	PA0727	A	G		hypothetical protein	
795424	SNV	PA0727	C	C		hypothetical protein	
795424	SNV	PA0727	C	T		hypothetical protein	
795667	SNV	PA0727	T	C		hypothetical protein	
795667	SNV	PA0727	T	T		hypothetical protein	
795671	SNV	PA0727	C	C		hypothetical protein	
795671	SNV	PA0727	C	T		hypothetical protein	
795742	SNV	PA0727	C	C		hypothetical protein	
795742	SNV	PA0727	C	T		hypothetical protein	
795745	SNV	PA0727	A	A		hypothetical protein	
795745	SNV	PA0727	A	C		hypothetical protein	
795754	SNV	PA0727	G	C		hypothetical protein	
795754	SNV	PA0727	G	G		hypothetical protein	
795766	SNV	PA0727	G	C		hypothetical protein	
795766	SNV	PA0727	G	G		hypothetical protein	
1587916	SNV	PA1458	T	C		two-component sensor	
1587919	SNV	PA1458	G	T	NP_250149.1:p.Glu299Asp	two-component sensor	
1587941	SNV	PA1458	T	G	NP_250149.1:p.Ser307Ala	two-component sensor	
1587943	SNV	PA1458	G	A		two-component sensor	
1856056	SNV	exsC	C	C		exoenzyme S synthesis protein C	
1856056	SNV	exsC	C	G		exoenzyme S synthesis protein C	
1856058	SNV	exsC	A	A		exoenzyme S synthesis protein C	
1856058	SNV	exsC	A	G	NP_250401.1:p.Glu66Gly	exoenzyme S synthesis protein C	
3230295	SNV	PA2877	A	A		transcriptional regulator	
3230295	SNV	PA2877	A	G	NP_251567.1:p.Leu293Pro	transcriptional regulator	
4145318	SNV	wspF	G	T	NP_252392.1:p.Ala210Glu	chemotaxis-specific methylesterase	

	4699910	Deletion		C	-			
	5242141	SNV		G	C			
	5242141	SNV		G	G			
	2753522^2753523	Insertion	PA2451	-	C			
	740419^740420	Insertion	PA0683	-	C	NP_249374.1:p.Val73fs	type II secretion system protein	
	789219..789220	MNV		GG	AT			
	789219..789220	MNV		GG	GG			
	790485..790486	MNV	PA0720	TG	CC	NP_249411.1:p.Met107Thr	helix destabilizing protein of bacteriophage Pf1	
	790485..790486	MNV	PA0720	TG	TG		helix destabilizing protein of bacteriophage Pf1	
	790521..790522	MNV	PA0720	TG	CA	NP_249411.1:p.Val119Ala	helix destabilizing protein of bacteriophage Pf1	
	790521..790522	MNV	PA0720	TG	TG		helix destabilizing protein of bacteriophage Pf1	
	794989..794990	MNV	PA0727	CT	CT		hypothetical protein	
	794989..794990	MNV	PA0727	CT	GC		hypothetical protein	
	795009..795010	MNV	PA0727	AA	AA		hypothetical protein	
	795009..795010	MNV	PA0727	AA	CG	NP_249418.1:p.Lys170Thr	hypothetical protein	
	795019^795020	Insertion	PA0727	-	-		hypothetical protein	
	795019^795020	Insertion	PA0727	-	T	NP_249418.1:p.Asp174fs	hypothetical protein	

RSCV 4

Region	Type	Gene	Original sequence	Sequence Change	Amino acid change	Functional Group
64793	SNV		A	A		
64793	SNV		A	G		
721670	SNV		A	G		
721718	SNV		A	G		
721725	SNV		C	T		
721740	SNV		C	T		
788573	SNV		T	C		
788573	SNV		T	T		
788581	SNV		G	G		
788581	SNV		G	T		
789170	SNV		C	C		
789170	SNV		C	T		

789185	SNV		A	A		
789185	SNV		A	C		
789266	SNV		C	C		
789266	SNV		C	T		
790701	SNV	PA0721	G	A	NP_249412.1:p.Ala29Thr	hypothetical protein
790701	SNV	PA0721	G	G		hypothetical protein
792124	SNV	PA0724	G	A	NP_249415.1:p.Gly252Asp	phage coat protein A
792124	SNV	PA0724	G	G		phage coat protein A
792443	SNV	PA0724	T	C		phage coat protein A
792443	SNV	PA0724	T	T		phage coat protein A
795025	SNV	PA0727	G	C		hypothetical protein
795025	SNV	PA0727	G	G		hypothetical protein
795028	SNV	PA0727	G	G		hypothetical protein
795028	SNV	PA0727	G	T		hypothetical protein
795385	SNV	PA0727	T	C		hypothetical protein
795385	SNV	PA0727	T	T		hypothetical protein
795391	SNV	PA0727	G	C		hypothetical protein
795391	SNV	PA0727	G	G		hypothetical protein
795403	SNV	PA0727	C	C		hypothetical protein
795403	SNV	PA0727	C	T		hypothetical protein
795406	SNV	PA0727	G	A		hypothetical protein
795406	SNV	PA0727	G	G		hypothetical protein
795409	SNV	PA0727	A	A		hypothetical protein
795409	SNV	PA0727	A	G		hypothetical protein
795412	SNV	PA0727	A	A		hypothetical protein
795412	SNV	PA0727	A	G		hypothetical protein
795424	SNV	PA0727	C	C		hypothetical protein
795424	SNV	PA0727	C	T		hypothetical protein
795432	Deletion	PA0727	A	-	NP_249418.1:p.Ser312fs	hypothetical protein
795432	SNV	PA0727	A	A		hypothetical protein

795434	SNV	PA0727	A	A		hypothetical protein	
795434	SNV	PA0727	A	G	NP_249418.1:p.Ser312Gly	hypothetical protein	
795667	SNV	PA0727	T	C		hypothetical protein	
795667	SNV	PA0727	T	T		hypothetical protein	
795671	SNV	PA0727	C	C		hypothetical protein	
795671	SNV	PA0727	C	T		hypothetical protein	
795742	SNV	PA0727	C	C		hypothetical protein	
795742	SNV	PA0727	C	T		hypothetical protein	
795745	SNV	PA0727	A	A		hypothetical protein	
795745	SNV	PA0727	A	C		hypothetical protein	
795754	SNV	PA0727	G	C		hypothetical protein	
795754	SNV	PA0727	G	G		hypothetical protein	
795766	SNV	PA0727	G	C		hypothetical protein	
795766	SNV	PA0727	G	G		hypothetical protein	
1587916	SNV	PA1458	T	C		two-component sensor	
1587919	SNV	PA1458	G	T	NP_250149.1:p.Glu299Asp	two-component sensor	
1587943	SNV	PA1458	G	A		two-component sensor	
1856056	SNV	exsC	C	C		exoenzyme S synthesis protein C	
1856056	SNV	exsC	C	G		exoenzyme S synthesis protein C	
1856058	SNV	exsC	A	A		exoenzyme S synthesis protein C	
1856058	SNV	exsC	A	G	NP_250401.1:p.Glu66Gly	exoenzyme S synthesis protein C	
3230295	SNV	PA2877	A	A		transcriptional regulator	
3230295	SNV	PA2877	A	G	NP_251567.1:p.Leu293Pro	transcriptional regulator	
4145042	SNV	wspF	C	T	NP_252392.1:p.Cys302Tyr	chemotaxis-specific methylesterase	
4699910	Deletion		C	-			
5242141	SNV		G	C			
5242141	SNV		G	G			

721663..721664	MNV		TC	CT		
789219..789220	MNV		GG	AT		
789219..789220	MNV		GG	GG		
794989..794990	MNV	PA0727	CT	CT		hypothetical protein
794989..794990	MNV	PA0727	CT	GC		hypothetical protein
795009..795010	MNV	PA0727	AA	AA		hypothetical protein
795009..795010	MNV	PA0727	AA	CG	NP_249418.1:p.Lys170Thr	hypothetical protein
795019^795020	Insertion	PA0727	-	-		hypothetical protein
795019^795020	Insertion	PA0727	-	T	NP_249418.1:p.Asp174fs	hypothetical protein
98901^98902	Insertion	fha1	-	-		Fha domain-containing protein
98901^98902	Insertion	fha1	-	TGGCTG	NP_248771.1:p.Pro285_Ala286insGlnPro	Fha domain-containing protein

RSCV 5

Region	Type	Gene	Original sequence	Sequence Change	Amino acid change	Functional Group
64793	SNV		A	A		
64793	SNV		A	G		
721670	SNV		A	G		
721718	SNV		A	G		
721725	SNV		C	T		
721740	SNV		C	T		
788573	SNV		T	C		
788573	SNV		T	T		
788581	SNV		G	G		
788581	SNV		G	T		
789170	SNV		C	C		

789170	SNV		C	T		
789185	SNV		A	A		
789185	SNV		A	C		
789266	SNV		C	C		
789266	SNV		C	T		
792608	SNV	PA0724	C	C		phage coat protein A
792608	SNV	PA0724	C	T		phage coat protein A
793757	SNV	PA0726	C	C		hypothetical protein
793757	SNV	PA0726	C	G	NP_249417.1:p.Ala254Gly	hypothetical protein
794001	SNV	PA0726	C	C		hypothetical protein
794001	SNV	PA0726	C	G	NP_249417.1:p.Asp335Glu	hypothetical protein
794106	SNV	PA0726	G	C	NP_249417.1:p.Glu370Asp	hypothetical protein
794106	SNV	PA0726	G	G		hypothetical protein
794140	SNV	PA0726	C	C		hypothetical protein
794140	SNV	PA0726	C	G	NP_249417.1:p.Pro382Ala	hypothetical protein
794195	SNV	PA0726	C	C		hypothetical protein
794195	SNV	PA0726	C	T	NP_249417.1:p.Ala400Val	hypothetical protein
794430	SNV		C	C		
794430	SNV		C	T		
794612	SNV	PA0727	C	C		hypothetical protein
794612	SNV	PA0727	C	T		hypothetical protein
794629	SNV	PA0727	T	C		hypothetical protein
794629	SNV	PA0727	T	T		hypothetical protein
794650	SNV	PA0727	G	C		hypothetical protein
794650	SNV	PA0727	G	G		hypothetical protein
794680	SNV	PA0727	G	C		hypothetical protein
794680	SNV	PA0727	G	G		hypothetical protein
795025	SNV	PA0727	G	C		hypothetical protein
795025	SNV	PA0727	G	G		hypothetical protein
795028	SNV	PA0727	G	G		hypothetical protein

795028	SNV	PA0727	G	T		hypothetical protein
795296	SNV	PA0727	C	A	NP_249418.1:p.Leu266Ile	hypothetical protein
795296	SNV	PA0727	C	C		hypothetical protein
795304	SNV	PA0727	C	C		hypothetical protein
795304	SNV	PA0727	C	T		hypothetical protein
795310	SNV	PA0727	C	C		hypothetical protein
795310	SNV	PA0727	C	T		hypothetical protein
795313	SNV	PA0727	T	C		hypothetical protein
795313	SNV	PA0727	T	T		hypothetical protein
795349	SNV	PA0727	C	C		hypothetical protein
795349	SNV	PA0727	C	T		hypothetical protein
795370	SNV	PA0727	T	C		hypothetical protein
795370	SNV	PA0727	T	T		hypothetical protein
795385	SNV	PA0727	T	C		hypothetical protein
795385	SNV	PA0727	T	T		hypothetical protein
795391	SNV	PA0727	G	C		hypothetical protein
795391	SNV	PA0727	G	G		hypothetical protein
795403	SNV	PA0727	C	C		hypothetical protein
795403	SNV	PA0727	C	T		hypothetical protein
795406	SNV	PA0727	G	A		hypothetical protein
795406	SNV	PA0727	G	G		hypothetical protein
795409	SNV	PA0727	A	A		hypothetical protein
795409	SNV	PA0727	A	G		hypothetical protein
795412	SNV	PA0727	A	A		hypothetical protein
795412	SNV	PA0727	A	G		hypothetical protein
795424	SNV	PA0727	C	C		hypothetical protein
795424	SNV	PA0727	C	T		hypothetical protein
795432	Deletion	PA0727	A	-	NP_249418.1:p.Ser312fs	hypothetical protein
795432	SNV	PA0727	A	A		hypothetical protein
795434	SNV	PA0727	A	A		hypothetical protein

795434	SNV	PA0727	A	G	NP_249418.1:p.Ser312Gly	hypothetical protein	
795667	SNV	PA0727	T	C		hypothetical protein	
795667	SNV	PA0727	T	T		hypothetical protein	
795671	SNV	PA0727	C	C		hypothetical protein	
795671	SNV	PA0727	C	T		hypothetical protein	
795742	SNV	PA0727	C	C		hypothetical protein	
795742	SNV	PA0727	C	T		hypothetical protein	
795745	SNV	PA0727	A	A		hypothetical protein	
795745	SNV	PA0727	A	C		hypothetical protein	
795754	SNV	PA0727	G	C		hypothetical protein	
795754	SNV	PA0727	G	G		hypothetical protein	
795766	SNV	PA0727	G	C		hypothetical protein	
795766	SNV	PA0727	G	G		hypothetical protein	
1587916	SNV	PA1458	T	C		two-component sensor	
1587919	SNV	PA1458	G	T	NP_250149.1:p.Glu299Asp	two-component sensor	
1587941	SNV	PA1458	T	G	NP_250149.1:p.Ser307Ala	two-component sensor	
1587943	SNV	PA1458	G	A		two-component sensor	
1856056	SNV	exsC	C	C		exoenzyme S synthesis protein C	
1856056	SNV	exsC	C	G		exoenzyme S synthesis protein C	
1856058	SNV	exsC	A	A		exoenzyme S synthesis protein C	
1856058	SNV	exsC	A	G	NP_250401.1:p.Glu66Gly	exoenzyme S synthesis protein C	
3230295	SNV	PA2877	A	A		transcriptional regulator	
3230295	SNV	PA2877	A	G	NP_251567.1:p.Leu293Pro	transcriptional regulator	
4145307	SNV	wspF	C	A	NP_252392.1:p.Glu214*	chemotaxis-specific methylesterase	
4699910	Deletion		C	-			
5242141	SNV		G	C			
5242141	SNV		G	G			

721663..721664	MNV		TC	CT		
789219..789220	MNV		GG	AT		
789219..789220	MNV		GG	GG		
794989..794990	MNV	PA0727	CT	CT		hypothetical protein
794989..794990	MNV	PA0727	CT	GC		hypothetical protein
795009..795010	MNV	PA0727	AA	AA		hypothetical protein
795009..795010	MNV	PA0727	AA	CG	NP_249418.1:p.Lys170Thr	hypothetical protein
795019^795020	Insertion	PA0727	-	-		hypothetical protein
795019^795020	Insertion	PA0727	-	T	NP_249418.1:p.Asp174fs	hypothetical protein
98901^98902	Insertion	fha1	-	-		Fha domain-containing protein
98901^98902	Insertion	fha1	-	TGGCTG	NP_248771.1:p.Pro285_Ala286insGlnPro	Fha domain-containing protein

RSCV 6

Region	Type	Gene	Original sequence	Sequence Change	Amino acid change	Functional Group
64793	SNV		A	A		
64793	SNV		A	G		
721670	SNV		A	G		
788573	SNV		T	C		
788573	SNV		T	T		
788581	SNV		G	G		
788581	SNV		G	T		
789170	SNV		C	C		
789170	SNV		C	T		
789185	SNV		A	A		

789185	SNV		A	C		
789266	SNV		C	C		
789266	SNV		C	T		
789288	SNV		C	C		
789288	SNV		C	T		
792359	SNV	PA0724	C	C		phage coat protein A
792359	SNV	PA0724	C	T		phage coat protein A
792401	SNV	PA0724	C	A	NP_249415.1:p.Asp344Glu	phage coat protein A
792401	SNV	PA0724	C	C		phage coat protein A
792443	SNV	PA0724	T	C		phage coat protein A
792443	SNV	PA0724	T	T		phage coat protein A
792455	SNV	PA0724	C	C		phage coat protein A
792455	SNV	PA0724	C	T		phage coat protein A
792590	SNV	PA0724	G	A		phage coat protein A
792590	SNV	PA0724	G	G		phage coat protein A
792608	SNV	PA0724	C	C		phage coat protein A
792608	SNV	PA0724	C	T		phage coat protein A
795025	SNV	PA0727	G	C		hypothetical protein
795025	SNV	PA0727	G	G		hypothetical protein
795028	SNV	PA0727	G	G		hypothetical protein
795028	SNV	PA0727	G	T		hypothetical protein
795667	SNV	PA0727	T	C		hypothetical protein
795667	SNV	PA0727	T	T		hypothetical protein
795671	SNV	PA0727	C	C		hypothetical protein
795671	SNV	PA0727	C	T		hypothetical protein
795742	SNV	PA0727	C	C		hypothetical protein
795742	SNV	PA0727	C	T		hypothetical protein
795745	SNV	PA0727	A	A		hypothetical protein
795745	SNV	PA0727	A	C		hypothetical protein
795754	SNV	PA0727	G	C		hypothetical protein

795754	SNV	PA0727	G	G		hypothetical protein	
795766	SNV	PA0727	G	C		hypothetical protein	
795766	SNV	PA0727	G	G		hypothetical protein	
1275767	Deletion	napA	A	-	NP_249865.1:p.Phe11fs	nitrate reductase catalytic subunit	
1587916	SNV	PA1458	T	C		two-component sensor	
1587919	SNV	PA1458	G	T	NP_250149.1:p.Glu299Asp	two-component sensor	
1587941	SNV	PA1458	T	G	NP_250149.1:p.Ser307Ala	two-component sensor	
1587943	SNV	PA1458	G	A		two-component sensor	
1856056	SNV	exsC	C	C		exoenzyme S synthesis protein C	
1856056	SNV	exsC	C	G		exoenzyme S synthesis protein C	
1856058	SNV	exsC	A	A		exoenzyme S synthesis protein C	
1856058	SNV	exsC	A	G	NP_250401.1:p.Glu66Gly	exoenzyme S synthesis protein C	
3230295	SNV	PA2877	A	A		transcriptional regulator	
3230295	SNV	PA2877	A	G	NP_251567.1:p.Leu293Pro	transcriptional regulator	
4145096	SNV	wspF	A	C	NP_252392.1:p.Leu284Arg	chemotaxis-specific methylesterase	
4699910	Deletion		C	-			
6132110	SNV	uvrD	C	A	NP_254130.1:p.Ser341Arg	DNA-dependent helicase II	
2195457^2195458	Insertion		-	C			
2753522^2753523	Insertion	PA2451	-	C			
5071544..5071547	Deletion		ACTG	-			
667028^667029	Insertion		-	C			
721663..721664	MNV		TC	CT			
789219..789220	MNV		GG	AT			
789219..789220	MNV		GG	GG			
794989..794990	MNV	PA0727	CT	CT		hypothetical protein	
794989..794990	MNV	PA0727	CT	GC		hypothetical protein	
795009..795010	MNV	PA0727	AA	AA		hypothetical protein	

795009..795010	MNV	PA0727	AA	CG	NP_249418.1:p.Lys170Thr	hypothetical protein
795019^795020	Insertion	PA0727	-	-		hypothetical protein
795019^795020	Insertion	PA0727	-	T	NP_249418.1:p.Asp174fs	hypothetical protein
816529^816530	Insertion	PA0748	-	C		

RSCV 7

Region	Type	Gene	Original sequence	Sequence Change	Amino acid change	Functional Group
64793	SNV		A	A		
64793	SNV		A	G		
788573	SNV		T	C		
788573	SNV		T	T		
788581	SNV		G	G		
788581	SNV		G	T		
789170	SNV		C	C		
789170	SNV		C	T		
789185	SNV		A	A		
789185	SNV		A	C		
789219..789220	MNV		GG	AT		

789219..789220	MNV		GG	GG		
795667	SNV	PA0727	T	C		hypothetical protein
795667	SNV	PA0727	T	T		hypothetical protein
795671	SNV	PA0727	C	C		hypothetical protein
795671	SNV	PA0727	C	T		hypothetical protein
795742	SNV	PA0727	C	C		hypothetical protein
795742	SNV	PA0727	C	T		hypothetical protein
795745	SNV	PA0727	A	A		hypothetical protein
795745	SNV	PA0727	A	C		hypothetical protein
795754	SNV	PA0727	G	C		hypothetical protein
795754	SNV	PA0727	G	G		hypothetical protein
795766	SNV	PA0727	G	C		hypothetical protein
795766	SNV	PA0727	G	G		hypothetical protein
1856056	SNV	exsC	C	C		exoenzyme S synthesis protein C
1856056	SNV	exsC	C	G		exoenzyme S synthesis protein C
1856058	SNV	exsC	A	A		exoenzyme S synthesis protein C
1856058	SNV	exsC	A	G	NP_250401.1:p.Glu66Gly	exoenzyme S synthesis protein C
3230295	SNV	PA2877	A	A		transcriptional regulator
3230295	SNV	PA2877	A	G	NP_251567.1:p.Leu293Pro	transcriptional regulator
4699910	Deletion		C	-		
721663..721664	MNV		TC	CT		
721670	SNV		A	G		
721718	SNV		A	G		
721725	SNV		C	T		
721740	SNV		C	T		
794989..794990	MNV	PA0727	CT	CT		hypothetical protein
794989..794990	MNV	PA0727	CT	GC		hypothetical protein

	795009..795010	MNV	PA0727	AA	AA		hypothetical protein	
	795009..795010	MNV	PA0727	AA	CG	NP_249418.1:p.Lys170Thr	hypothetical protein	
	795019^795020	Insertion	PA0727	-	-		hypothetical protein	
	795019^795020	Insertion	PA0727	-	T	NP_249418.1:p.Asp174fs	hypothetical protein	
	795025	SNV	PA0727	G	C		hypothetical protein	
	795025	SNV	PA0727	G	G		hypothetical protein	
	795028	SNV	PA0727	G	G		hypothetical protein	
	795028	SNV	PA0727	G	T		hypothetical protein	
	795385	SNV	PA0727	T	C		hypothetical protein	
	795385	SNV	PA0727	T	T		hypothetical protein	
	795391	SNV	PA0727	G	C		hypothetical protein	
	795391	SNV	PA0727	G	G		hypothetical protein	
	795403	SNV	PA0727	C	C		hypothetical protein	
	795403	SNV	PA0727	C	T		hypothetical protein	
	795406	SNV	PA0727	G	A		hypothetical protein	
	795406	SNV	PA0727	G	G		hypothetical protein	
	795409	SNV	PA0727	A	A		hypothetical protein	
	795409	SNV	PA0727	A	G		hypothetical protein	
	795412	SNV	PA0727	A	A		hypothetical protein	
	795412	SNV	PA0727	A	G		hypothetical protein	
	795424	SNV	PA0727	C	C		hypothetical protein	
	795424	SNV	PA0727	C	T		hypothetical protein	
	795432	Deletion	PA0727	A	-	NP_249418.1:p.Ser312fs	hypothetical protein	
	795432	SNV	PA0727	A	A		hypothetical protein	
	795434	SNV	PA0727	A	A		hypothetical protein	
	795434	SNV	PA0727	A	G	NP_249418.1:p.Ser312Gly	hypothetical protein	
	795472	SNV	PA0727	G	C		hypothetical protein	
	795472	SNV	PA0727	G	G		hypothetical protein	
	4145114..4145117	Deletion	wspF	CGGC	-	NP_252392.1:p.Gly277fs	chemotaxis-specific methylesterase	

RSCV 8

Region	Type	Gene	Original sequence	Sequence Change	Amino acid change	Functional Group
64793	SNV		A	A		
64793	SNV		A	G		
721670	SNV		A	G		
721725	SNV		C	T		
788573	SNV		T	C		
788573	SNV		T	T		
788581	SNV		G	G		
788581	SNV		G	T		
789170	SNV		C	C		
789170	SNV		C	T		
789185	SNV		A	A		
789185	SNV		A	C		

789266	SNV		C	C			
789266	SNV		C	T			
789288	SNV		C	C			
789288	SNV		C	T			
789332	SNV		C	C			
789332	SNV		C	T			
789409	SNV	PA0718	C	C		hypothetical protein	
789409	SNV	PA0718	C	G	NP_249409.1:p.Ala17Gly	hypothetical protein	
789439	SNV	PA0718	T	C	NP_249409.1:p.Val27Ala	hypothetical protein	
789439	SNV	PA0718	T	T		hypothetical protein	
789470	SNV	PA0718	A	A		hypothetical protein	
789470	SNV	PA0718	A	G		hypothetical protein	
789473	SNV	PA0718	C	C		hypothetical protein	
789473	SNV	PA0718	C	T		hypothetical protein	
789476	SNV	PA0718	T	C		hypothetical protein	
789476	SNV	PA0718	T	T		hypothetical protein	
789505	SNV	PA0718	G	A	NP_249409.1:p.Gly49Asp	hypothetical protein	
789505	SNV	PA0718	G	G		hypothetical protein	
789602	SNV	PA0718	G	A		hypothetical protein	
789602	SNV	PA0718	G	G		hypothetical protein	
794806	SNV	PA0727	T	C		hypothetical protein	
794806	SNV	PA0727	T	T		hypothetical protein	
794827	SNV	PA0727	T	C		hypothetical protein	
794827	SNV	PA0727	T	T		hypothetical protein	
794962	SNV	PA0727	A	A		hypothetical protein	
794962	SNV	PA0727	A	G		hypothetical protein	
794974	SNV	PA0727	C	A		hypothetical protein	
794974	SNV	PA0727	C	C		hypothetical protein	
794990	SNV	PA0727	T	C		hypothetical protein	
794990	SNV	PA0727	T	T		hypothetical protein	

795031	SNV	PA0727	C	C		hypothetical protein
795031	SNV	PA0727	C	T		hypothetical protein
795058	SNV	PA0727	G	A		hypothetical protein
795058	SNV	PA0727	G	G		hypothetical protein
795088	SNV	PA0727	A	A		hypothetical protein
795088	SNV	PA0727	A	G		hypothetical protein
795385	SNV	PA0727	T	C		hypothetical protein
795385	SNV	PA0727	T	T		hypothetical protein
795391	SNV	PA0727	G	C		hypothetical protein
795391	SNV	PA0727	G	G		hypothetical protein
795403	SNV	PA0727	C	C		hypothetical protein
795403	SNV	PA0727	C	T		hypothetical protein
795406	SNV	PA0727	G	A		hypothetical protein
795406	SNV	PA0727	G	G		hypothetical protein
795409	SNV	PA0727	A	A		hypothetical protein
795409	SNV	PA0727	A	G		hypothetical protein
795412	SNV	PA0727	A	A		hypothetical protein
795412	SNV	PA0727	A	G		hypothetical protein
795424	SNV	PA0727	C	C		hypothetical protein
795424	SNV	PA0727	C	T		hypothetical protein
795432	Deletion	PA0727	A	-	NP_249418.1:p.Ser312fs	hypothetical protein
795432	SNV	PA0727	A	A		hypothetical protein
795434	SNV	PA0727	A	A		hypothetical protein
795434	SNV	PA0727	A	G	NP_249418.1:p.Ser312Gly	hypothetical protein
795472	SNV	PA0727	G	C		hypothetical protein
795472	SNV	PA0727	G	G		hypothetical protein
795742	SNV	PA0727	C	C		hypothetical protein
795742	SNV	PA0727	C	T		hypothetical protein
795745	SNV	PA0727	A	A		hypothetical protein
795745	SNV	PA0727	A	C		hypothetical protein

795754	SNV	PA0727	G	C		hypothetical protein
795754	SNV	PA0727	G	G		hypothetical protein
795766	SNV	PA0727	G	C		hypothetical protein
795766	SNV	PA0727	G	G		hypothetical protein
1587916	SNV	PA1458	T	C		two-component sensor
1587919	SNV	PA1458	G	T	NP_250149.1:p.Glu299Asp	two-component sensor
1856056	SNV	exsC	C	C		exoenzyme S synthesis protein C
1856056	SNV	exsC	C	G		exoenzyme S synthesis protein C
1856058	SNV	exsC	A	A		exoenzyme S synthesis protein C
1856058	SNV	exsC	A	G	NP_250401.1:p.Glu66Gly	exoenzyme S synthesis protein C
3230295	SNV	PA2877	A	A		transcriptional regulator
3230295	SNV	PA2877	A	G	NP_251567.1:p.Leu293Pro	transcriptional regulator
4145307	SNV	wspF	C	A	NP_252392.1:p.Glu214*	chemotaxis-specific methylesterase
4699910	Deletion		C	-		
5242141	SNV		G	C		
5242141	SNV		G	G		
721663..721664	MNV		TC	CT		
789219..789220	MNV		GG	AT		
789219..789220	MNV		GG	GG		

RSCV 9

Region	Type	Gene	Original sequence	Sequence Change	Amino acid change	Functional Group
64793	SNV		A	A		
64793	SNV		A	G		
721670	SNV		A	G		
721718	SNV		A	G		
721725	SNV		C	T		
721740	SNV		C	T		
788573	SNV		T	C		
788573	SNV		T	T		
788581	SNV		G	G		
788581	SNV		G	T		
789170	SNV		C	C		
789170	SNV		C	T		

789185	SNV		A	A		
789185	SNV		A	C		
789266	SNV		C	C		
789266	SNV		C	T		
789288	SNV		C	C		
789288	SNV		C	T		
789332	SNV		C	C		
789332	SNV		C	T		
789409	SNV	PA0718	C	C		hypothetical protein
789409	SNV	PA0718	C	G	NP_249409.1:p.Ala17Gly	hypothetical protein
789439	SNV	PA0718	T	C	NP_249409.1:p.Val27Ala	hypothetical protein
789439	SNV	PA0718	T	T		hypothetical protein
789470	SNV	PA0718	A	A		hypothetical protein
789470	SNV	PA0718	A	G		hypothetical protein
789473	SNV	PA0718	C	C		hypothetical protein
789473	SNV	PA0718	C	T		hypothetical protein
789476	SNV	PA0718	T	C		hypothetical protein
789476	SNV	PA0718	T	T		hypothetical protein
789505	SNV	PA0718	G	A	NP_249409.1:p.Gly49Asp	hypothetical protein
789505	SNV	PA0718	G	G		hypothetical protein
793996	SNV	PA0726	C	C		hypothetical protein
793996	SNV	PA0726	C	T		hypothetical protein
794430	SNV		C	C		
794430	SNV		C	T		
795025	SNV	PA0727	G	C		hypothetical protein
795025	SNV	PA0727	G	G		hypothetical protein
795028	SNV	PA0727	G	G		hypothetical protein
795028	SNV	PA0727	G	T		hypothetical protein
795385	SNV	PA0727	T	C		hypothetical protein
795385	SNV	PA0727	T	T		hypothetical protein

795391	SNV	PA0727	G	C		hypothetical protein	
795391	SNV	PA0727	G	G		hypothetical protein	
795403	SNV	PA0727	C	C		hypothetical protein	
795403	SNV	PA0727	C	T		hypothetical protein	
795406	SNV	PA0727	G	A		hypothetical protein	
795406	SNV	PA0727	G	G		hypothetical protein	
795409	SNV	PA0727	A	A		hypothetical protein	
795409	SNV	PA0727	A	G		hypothetical protein	
795412	SNV	PA0727	A	A		hypothetical protein	
795412	SNV	PA0727	A	G		hypothetical protein	
795667	SNV	PA0727	T	C		hypothetical protein	
795667	SNV	PA0727	T	T		hypothetical protein	
795671	SNV	PA0727	C	C		hypothetical protein	
795671	SNV	PA0727	C	T		hypothetical protein	
795742	SNV	PA0727	C	C		hypothetical protein	
795742	SNV	PA0727	C	T		hypothetical protein	
795745	SNV	PA0727	A	A		hypothetical protein	
795745	SNV	PA0727	A	C		hypothetical protein	
795754	SNV	PA0727	G	C		hypothetical protein	
795754	SNV	PA0727	G	G		hypothetical protein	
795766	SNV	PA0727	G	C		hypothetical protein	
795766	SNV	PA0727	G	G		hypothetical protein	
1587916	SNV	PA1458	T	C		two-component sensor	
1587919	SNV	PA1458	G	T	NP_250149.1:p.Glu299Asp	two-component sensor	
1587941	SNV	PA1458	T	G	NP_250149.1:p.Ser307Ala	two-component sensor	
1587943	SNV	PA1458	G	A		two-component sensor	
1856056	SNV	exsC	C	C		exoenzyme S synthesis protein C	
1856056	SNV	exsC	C	G		exoenzyme S synthesis protein C	
1856058	SNV	exsC	A	A		exoenzyme S synthesis	

						protein C
1856058	SNV	exsC	A	G	NP_250401.1:p.Glu66Gly	exoenzyme S synthesis protein C
3230295	SNV	PA2877	A	A		transcriptional regulator
3230295	SNV	PA2877	A	G	NP_251567.1:p.Leu293Pro	transcriptional regulator
4145058	SNV	wspF	G	A	NP_252392.1:p.Gln297*	chemotaxis-specific methylesterase
4699910	Deletion		C	-		
5242141	SNV		G	C		
5242141	SNV		G	G		
721663..721664	MNV		TC	CT		
789219..789220	MNV		GG	AT		
789219..789220	MNV		GG	GG		
794290..794291	MNV		CC	CC		
794290..794291	MNV		CC	TT		
794989..794990	MNV	PA0727	CT	CT		hypothetical protein
794989..794990	MNV	PA0727	CT	GC		hypothetical protein
795009..795010	MNV	PA0727	AA	AA		hypothetical protein
795009..795010	MNV	PA0727	AA	CG	NP_249418.1:p.Lys170Thr	hypothetical protein
795019^795020	Insertion	PA0727	-	-		hypothetical protein
795019^795020	Insertion	PA0727	-	T	NP_249418.1:p.Asp174fs	hypothetical protein

RSCV 10

Region	Type	Gene	Original sequence	Sequence Change	Amino acid change	Functional Group
64793	SNV		A	A		
64793	SNV		A	G		
721670	SNV		A	G		
721718	SNV		A	G		
721725	SNV		C	T		
721740	SNV		C	T		
788573	SNV		T	C		
788573	SNV		T	T		
788581	SNV		G	G		
788581	SNV		G	T		
789170	SNV		C	C		
789170	SNV		C	T		

789185	SNV		A	A		
789185	SNV		A	C		
789266	SNV		C	C		
789266	SNV		C	T		
789288	SNV		C	C		
789288	SNV		C	T		
789332	SNV		C	C		
789332	SNV		C	T		
789409	SNV	PA0718	C	C		hypothetical protein
789409	SNV	PA0718	C	G	NP_249409.1:p.Ala17Gly	hypothetical protein
795025	SNV	PA0727	G	C		hypothetical protein
795025	SNV	PA0727	G	G		hypothetical protein
795028	SNV	PA0727	G	G		hypothetical protein
795028	SNV	PA0727	G	T		hypothetical protein
795385	SNV	PA0727	T	C		hypothetical protein
795385	SNV	PA0727	T	T		hypothetical protein
795391	SNV	PA0727	G	C		hypothetical protein
795391	SNV	PA0727	G	G		hypothetical protein
795403	SNV	PA0727	C	C		hypothetical protein
795403	SNV	PA0727	C	T		hypothetical protein
795406	SNV	PA0727	G	A		hypothetical protein
795406	SNV	PA0727	G	G		hypothetical protein

795409	SNV	PA0727	A	A		hypothetical protein
795409	SNV	PA0727	A	G		hypothetical protein
795412	SNV	PA0727	A	A		hypothetical protein
795412	SNV	PA0727	A	G		hypothetical protein
795424	SNV	PA0727	C	C		hypothetical protein
795424	SNV	PA0727	C	T		hypothetical protein
795432	Deletion	PA0727	A	-	NP_249418.1:p.Ser312fs	hypothetical protein
795432	SNV	PA0727	A	A		hypothetical protein
795434	SNV	PA0727	A	A		hypothetical protein
795434	SNV	PA0727	A	G	NP_249418.1:p.Ser312Gly	hypothetical protein
795472	SNV	PA0727	G	C		hypothetical protein
795472	SNV	PA0727	G	G		hypothetical protein
795745	SNV	PA0727	A	A		hypothetical protein
795745	SNV	PA0727	A	C		hypothetical protein
795754	SNV	PA0727	G	C		hypothetical protein
795754	SNV	PA0727	G	G		hypothetical protein
795766	SNV	PA0727	G	C		hypothetical protein
795766	SNV	PA0727	G	G		hypothetical protein
1587916	SNV	PA1458	T	C		two-component sensor
1587919	SNV	PA145	G	T	NP_250149.1:p.Glu299Asp	two-component sensor

		8				
1587941	SNV	PA1458	T	G	NP_250149.1:p.Ser307Ala	two-component sensor
1587943	SNV	PA1458	G	A		two-component sensor
1856056	SNV	exsC	C	C		exoenzyme S synthesis protein C
1856056	SNV	exsC	C	G		exoenzyme S synthesis protein C
1856058	SNV	exsC	A	A		exoenzyme S synthesis protein C
1856058	SNV	exsC	A	G	NP_250401.1:p.Glu66Gly	exoenzyme S synthesis protein C
3230295	SNV	PA2877	A	A		transcriptional regulator
3230295	SNV	PA2877	A	G	NP_251567.1:p.Leu293Pro	transcriptional regulator
4699910	Deletion		C	-		
4144991..4145002	Deletion	wspF	TGCACCGCTGCA	-	NP_252392.1:p.Asp315_Gln319delinsGlu	chemotaxis-specific methylesterase
721663..721664	MNV		TC	CT		
789219..789220	MNV		GG	AT		
789219..789220	MNV		GG	GG		
794989..794990	MNV	PA0727	CT	CT		hypothetical protein
794989..794990	MNV	PA0727	CT	GC		hypothetical protein
795009..795010	MNV	PA0727	AA	AA		hypothetical protein
795009..795010	MNV	PA0727	AA	CG	NP_249418.1:p.Lys170Thr	hypothetical protein
795019^795020	Insertion	PA0727	-	-		hypothetical protein
795019^795020	Insertion	PA0727	-	T	NP_249418.1:p.Asp174fs	hypothetical protein

References

1 Holloway, B. W. & Morgan, A. F. Genome organization in Pseudomonas. *Annual review of microbiology* **40**, 79-105, doi:10.1146/annurev.mi.40.100186.000455 (1986).

2 Rybtke, M. T. *et al.* Fluorescence-based reporter for gauging cyclic di-GMP levels in Pseudomonas aeruginosa. *Appl Environ Microbiol* **78**, 5060-5069, doi:10.1128/AEM.00414-12 (2012).

3 Chen, Y. *et al.* Multiple diguanylate cyclase-coordinated regulation of pyoverdine synthesis in Pseudomonas aeruginosa. *Environmental microbiology reports* **7**, 498-507, doi:10.1111/1758-2229.12278 (2015).

4 Irie, Y. *et al.* Self-produced exopolysaccharide is a signal that stimulates biofilm formation in Pseudomonas aeruginosa. *Proc Natl Acad Sci U S A* **109**, 20632-20636, doi:10.1073/pnas.1217993109 (2012).

5 Chua, S. L. *et al.* Bis-(3'-5')-cyclic dimeric GMP regulates antimicrobial peptide resistance in Pseudomonas aeruginosa. *Antimicrobial agents and chemotherapy* **57**, 2066-2075, doi:10.1128/AAC.02499-12 (2013).

6 Yang, L. *et al.* Distinct roles of extracellular polymeric substances in Pseudomonas aeruginosa biofilm development. *Environ Microbiol* **13**, 1705-1717, doi:10.1111/j.1462-2920.2011.02503.x (2011).

7 Carlier, A. *et al.* Genome Sequence of Burkholderia cenocepacia H111, a Cystic Fibrosis Airway Isolate. *Genome announcements* **2**, doi:10.1128/genomeA.00298-14 (2014).

8 Lee, K. W. *et al.* Biofilm development and enhanced stress resistance of a model, mixed-species community biofilm. *The ISME journal* **8**, 894-907, doi:10.1038/ismej.2013.194 (2014).

9 Gjermansen, M., Nilsson, M., Yang, L. & Tolker-Nielsen, T. Characterization of starvation-induced dispersion in Pseudomonas putida biofilms: genetic elements and molecular mechanisms. *Molecular microbiology* **75**, 815-826, doi:10.1111/j.1365-2958.2009.06793.x (2010).

10 Kessler, B., de Lorenzo, V. & Timmis, K. N. A general system to integrate lacZ fusions into the chromosomes of gram-negative eubacteria: regulation of the Pm promoter of the TOL plasmid studied with all controlling elements in monocopy. *Molecular & general genetics : MGG* **233**, 293-301 (1992).
